# Nutritional and Nutraceutical Support to the Failing Myocardium: A Possible Way of Potentiating the Current Treatment of Heart Failure

**DOI:** 10.3390/ijms252212232

**Published:** 2024-11-14

**Authors:** Roberta Macrì, Rocco Mollace, Maria Serra, Federica Scarano, Giovanna Ritorto, Sara Ussia, Antonio Cardamone, Anna Rita Coppoletta, Cristina Carresi, Micaela Gliozzi, Vincenzo Musolino, Jessica Maiuolo, Ernesto Palma, Maurizio Volterrani, Vincenzo Mollace, Carolina Muscoli

**Affiliations:** 1 Pharmacology Laboratory, Institute of Research for Food Safety and Health IRC-FSH, Department of Health Sciences, University Magna Graecia of Catanzaro, 88100 Catanzaro, Italy; maria.serra@studenti.unicz.it (M.S.); federicascar87@gmail.com (F.S.); giovanna.ritorto@studenti.unicz.it (G.R.); saraussia1598@gmail.com (S.U.); tony.c@outlook.it (A.C.); annarita.coppoletta@libero.it (A.R.C.); gliozzi@unicz.it (M.G.); mollace@libero.it (V.M.); muscoli@unicz.it (C.M.); 2 Department of Systems Medicine, University “Tor Vergata” of Rome, 00133 Rome, Italy; 3 Veterinary Pharmacology Laboratory, Institute of Research for Food Safety and Health IRC-FSH, Department of Health Sciences, University Magna Graecia of Catanzaro, 88100 Catanzaro, Italy; carresi@unicz.it (C.C.); palma@unicz.it (E.P.); 4 Laboratory of Pharmaceutical Biology, IRC-FSH Department of Health Sciences, University “Magna Græcia” of Catanzaro, Campus Universitario di Germaneto, 88100 Catanzaro, Italy; v.musolino@unicz.it (V.M.); maiuolo@unicz.it (J.M.); 5 IRCCS San Raffaele Roma, 00163 Rome, Italy; maurizio.volterrani@sanraffaele.it; 6 Renato Dulbecco Institute, Lamezia Terme, 88046 Catanzaro, Italy

**Keywords:** heart failure, lifestyle modifications, nutraceutical supplementation, phytochemical derivatives

## Abstract

Heart failure (HF) is a complex condition that affects 1–2% of the global population. The presence of comorbidities like diabetes, hypertension, hyperlipidemia, or obesity has been shown in various studies to elevate mortality and hospitalization rates in HF patients. Insufficient outcomes persist in HF, necessitating additional research to address unmet needs in disease management. Lifestyle modifications, including smoking cessation, decreased alcohol consumption, regular exercise, cardiac rehabilitation, and a balanced diet, can prevent and treat a wide range of HF cases. In this review, we aimed to examine how lifestyle changes, nutrition, and nutraceutical supplements can play a role in preventing heart failure and supporting its treatment. A detailed and comprehensive analysis of the most recent data present in the literature could help identify potential candidates for future clinical trials in HF management. There is a growing body of evidence supporting the importance of closely monitoring nutritional balance, including micronutrients and nutraceuticals, in HF patients for better symptom management and outcomes. Despite promising results from initial approaches, the lack of conclusive evidence from recent studies and meta-analyses questions the widespread use of nutraceutical supplementation in HF patients. Further studies are necessary to determine the most effective way to use nutraceutical supplementation in the treatment of myocardial dysfunction in HF patients.

## 1. Introduction

### 1.1. Incidence of Heart Failure (HF) and Pathophysiological Mechanisms

Heart failure (HF) is a complex condition that affects 1–2% of the global population, and extensive research has been conducted on it [1]. Several studies indicate that the presence of additional diseases like diabetes, hypertension, hyperlipidemia, or obesity can raise mortality and hospitalization rates in HF patients [2]. Age is a risk factor for patients, with a 10% increase in occurrence for those over 70 [3,4]. Recent international guidelines have demonstrated that better management of risk factors and increased treatment options can significantly decrease the impact of heart failure and its consequences [5]. The European Society of Cardiology (ESC) recently conducted a pilot study on HF and discovered a high prevalence of all-cause mortality. Over the course of 12 months, 17 HF patients were admitted to the hospital, with hospitalization rates of 44% and 32% for stable/ambulatory patients [6]. There are still unsatisfactory outcomes in relation to HF, and more studies are needed to address the unmet needs in managing the disease. Growing evidence supports closely monitoring nutritional balance, including micronutrients and nutraceuticals, in HF patients, resulting in improved symptoms and outcomes of the illness. However, there is limited proof regarding the link between lifestyle changes and HF pathology. This issue requires additional clinical studies for clarification [7,8]. It is essential to investigate new biomolecular mechanisms that trigger HF despite existing therapies, which highlights the potential role of nutrition in managing the condition [9].

### 1.2. Micronutrient Depletion in HF: Nutrition and Nutraceutical Supplementation

Insufficient micronutrient support among HF patients is indicated as the cause of increased disease prevalence. Moreover, conditions of impaired muscle function and a malfunctioning myocardium could be connected to oxidative stress and lack of micronutrients [10]. Evidence has demonstrated that an inadequate micronutrient supply can cause HF and exacerbate already existing cardiac dysfunction. Various conditions related to micronutrient deficiency can help prevent the loss of lean body mass (LBM) [11].

The causes of heart failure have been clarified, encompassing a range of factors like increased stress on the heart, dysfunction due to lack of blood flow, remodeling of the heart’s chambers, excessive activation of hormones, irregular movement of calcium in heart cells, abnormal buildup of material around cells, increased cell death, and genetic mutations [12]. While there is not one specific theory that can completely clarify HF, there is evidence pointing towards the importance of declining bioenergy [13]. Cardiomyocytes primarily rely on fatty acids and carbohydrates for energy. Micronutrients like coenzyme Q10, thiamine, riboflavin, etc., are vital cofactors needed for the conversion of macronutrients into biological energy, facilitating energy production, transfer, and maintaining heart function (Table 1) [14]. Additionally, individuals with HF experience oxidative stress, improper dietary habits, changes in metabolism and gut function, and inflammation, resulting in a shortage of essential nutrients (such as iron, selenium, and zinc) that impacts their outlook [15].

The functions controlled by vitamin D in the progression of HF include cell differentiation, oxidative stress, inflammation, and vascular calcification [16]. Observational research has indicated a correlation between low vitamin D levels and a higher likelihood of developing HF. Vitamin D deficiency is a prevalent type of hypovitaminosis globally, affecting nearly half of the elderly population, and is the most widespread vitamin deficiency in HF [17].

Vitamin D is linked to functional status, severity of illness, and outlook in HF [18]. The level of vitamin D is linked to improved results in cardiopulmonary stress tests and longer distances in the six-minute walking test, but is negatively related to NYHA class. A study indicated a significant difference in vitamin D levels between severe HF patients admitted to the hospital for intravenous inotropic agents or left ventricular assist devices and those managed as outpatients [19]. Vitamin D levels are linked to NT-proANP, NT-proBNP, and left ventricle ejection fraction (LVEF), which are indicators of prognosis in HF.

Coenzyme Q10 (CoQ10) plays a crucial role in ATP production and serves as a strong antioxidant that can dissolve in lipids. Patients with HF have shown a deficiency in myocardial CoQ10, which is linked to the severity of symptoms and LVEF. In elderly patients and individuals with advanced HF, lower levels of CoQ10 are detected [20].

Iron deficiency is a prevalent comorbidity in HF, impacting 37–61% of patients [21]. Iron deficiency in CHF patients, before anemia develops, can be significant, impacting symptoms, quality of life, and mortality rates [21]. According to the ESC guidelines, it is recommended that all HF patients be screened for iron deficiency. Iron deficiency is common in HFpEF, leading to decreased exercise capacity and quality of life, and becomes more prevalent as diastolic dysfunction worsens [22].

Early findings suggest that nutraceutical supplementation may be helpful for early-stage HF patients, but recent studies show insufficient evidence to recommend its widespread use in HF patients [23,24]. Consequently, even though nutraceutical supplementation appears promising for preventing and treating cardiovascular disease, additional evaluation is essential to assess its potential for treating HF [25].

Therefore, the purpose of this narrative review is to combine insights from clinical trials, systematic reviews, and meta-analyses from different research databases, including PubMed and Medline, that involved in vitro and in vivo studies and clinical trials to provide an overview of how lifestyle changes, nutrition, and nutraceutical supplements can prevent and support the treatment of heart failure. It also aims to identify potential candidates for future clinical studies in HF patients.

**Table 1:** Micronutrient depletion in HF.

Micronutrient Depletion in HF		
Clinical Trials	Properties	Ref.
An inadequate micronutrient supply	Cause HF and exacerbate cardiac dysfunction; malfunctioning myocardium could be connected to oxidative stress	[11]
Individuals with HF experience oxidative stress, improper dietary habits, changes in metabolism and gut function, and inflammation	Resulting in a shortage of essential nutrients (such as iron, selenium, and zinc) that impacts their outlook	[15]
Low vitamin D levels	Higher likelihood of developing HF	[17]
Level of vitamin D	Improved results in cardiopulmonary stress tests and longer distances in the six-minute walking test, but is negatively related to NYHA class Indicated a significant difference in vitamin D levels between severe HF patients admitted to the hospital for intravenous inotropic agents or left ventricular assist devices and those managed as outpatients	[19]
Patients with HF have shown a deficiency in myocardial CoQ10	Linked to the severity of symptoms and LVEF.	[20]
Iron deficiency	Impacting 37–61% of patients, can be significant in CHF patients before anemia development impacting symptoms, quality of life, and mortality rates	[21]
Iron deficiency is common in HFpEF	Decreased exercise capacity and quality of life, and becoming more prevalent as diastolic dysfunction worsens	[22]

HF: heart failure, NYHA: New York Heart Association, CoQ10: coenzyme q10, LVEF: left ventricular ejection fraction, CHF: chronic heart failure, HFpEF: heart failure with preserved ejection fraction.

## 2. Lifestyle Modifications in HF Management

**Figure 1:**
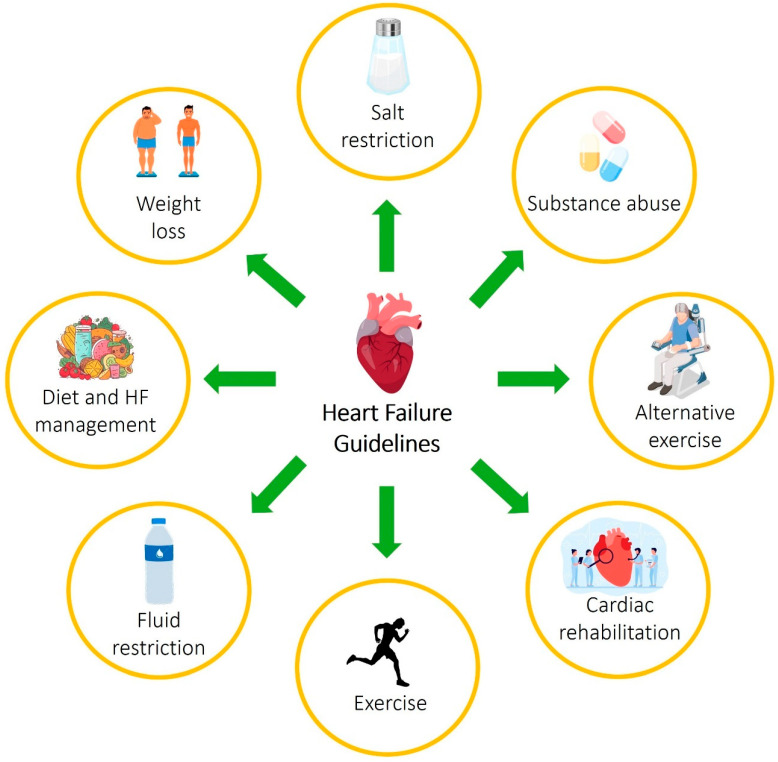
According to HF guidelines, the risk of heart failure can be reduced by minimizing salt consumption, substance abuse, fluid restriction, and weight loss through healthy eating, exercise, alternative exercise, and cardiac rehabilitation.

Heart failure (HF) is a complex and increasingly prevalent disease worldwide, resulting in significant mortality and morbidity, as advised by guidelines based on randomized clinical trials (RCTs) and various treatments [7]. HF guidelines are designed to aid clinicians in utilizing the most up-to-date research to treat HF patients effectively. Consequently, HF management relies on non-pharmacological and pharmacological approaches. Treatment with drugs is successful in reducing HF-related mortality and hospitalization (Figure 1) [26].

In terms of non-pharmacological treatment, the guidelines only suggest specific and artificial measures: avoiding excessive salt, maintaining a healthy weight, and increasing fluid intake. To improve quality of life (QoL), increase physical activity, enhance physiological conditions, and decrease hospitalizations for heart failure, exercise is recommended (Table 2) [27]. Elderly patients are frequently classified as extremely fragile individuals. Frailty, a geriatric clinical syndrome, is characterized by cachexia, a condition of widespread tissue wasting. It is common for elderly patients to encounter disability, challenges with physical activity, and metabolic disorders. Other chronic conditions like depression, anxiety, chronic obstructive pulmonary disease (COPD), and heart failure are usually associated with these. Frailty is accompanied by reduced antioxidant activity and heightened oxidative stress expression [28]. Lifestyle correction is necessary for elderly frail patients, who are seeing an increase in numbers [29,30]. A study investigating the influence of ET during exercise on sensitive and non-sensitive individuals found that frail individuals had a shorter 6 WMD at the start, but showed an equal increase after six months. There is a lack of findings regarding the effects of fluid or salt reduction. Nevertheless, patients with frail conditions may not be able to endure rigorous restrictions due to potential side effects such as compromised renal function or hyponatremia [31].

### 2.1. Exercise

#### 2.1.1. Exercise and HF Management

Physical activity is recommended by the guidelines as part of non-pharmacological treatment for HF, but unfortunately, inaccurately [55]. According to the Korean society of Heart Failure (KSHF) guidelines, it is recommended that exercise-related activities be supported and involve a specialized professional in the recovery of HF patients [56]. To enhance exercise capacity, decrease hospitalizations, and improve quality of life, the ESC clinical practice guidelines suggest physical activity for all eligible patients. Moreover, it is recommended that a cardiac rehabilitation program that prioritizes physical activity for patients with severe illnesses or fragility [32] be explored. The AHA/ACC HF 2022 guidelines also suggest that individuals with HF should engage in an exercise program to enhance their physical evidence, functional status, and quality of life [33,34].

##### Exercise Training (ET)

Currently, there are no clinical trials that have explored the effects of exercise training (ET) on subjects with varying phenotypes of heart failure with preserved ejection fraction (HFpEF) and heart failure with reduced ejection fraction (HFrEF), despite noteworthy results in previous clinical studies. The research analyzed 11,081 sample patients from 93 studies (82 HfrEF and 11 HFpEF). Health-related quality of life and exercise ability showed significant improvement with ET in both patient phenotypes. Specifically, in the 6 min walking distance (6 MWD), an increase in maximal oxygen consumption (VO2) (weighted mean change: 2.333 mL-min^−1^-kg^−1^, fixed *p* < 0.001) and E/e’ (mean change: 1.709 m) was highlighted. Nonetheless, there was no observable decline in hospital admissions or mortality after a short period [57]. Another study revealed that ET boosts exercise effectiveness, leading to a 15–17% improvement in the highest VO2. Several systematic reviews found that exercise time, workload, quality of life, and travel distance all increased in just 6 min [35]. The effectiveness of ET and rehabilitation on heart function and pulmonary circulation measurements was investigated in patients suffering from heart failure with preserved ejection fraction (HFpEF) through a meta-analysis of 18 articles. The post-training period showed a significant decrease in the mitral E/e ratio, as indicated by five clinical studies. The effects of ET on cardiac output, E-wave deceleration time (DecT), and E/A ratio were not clear, highlighting the need for more comprehensive studies to evaluate its effectiveness in cardiac function improvement [36].

On the other hand, a meta-analysis found that ET led to improved left ventricular ejection fraction (LVEF) in individuals with persistent HFrEF, especially during longer training sessions (≥6 months) [35]. In the EJECTION-HF study, a secondary analysis explored the connection between ET and increased 6 min walking distance, demonstrating enhanced frailty index (FI) and physical exercise for individuals with greater frailty. Death or readmission after 12 months was unrelated to the initial frailty or the intervention. Several limitations were present in this study. The analysis involved a pragmatic trial where a specific group was chosen despite open eligibility criteria, possibly impacting its generalizability. Elderly individuals, females, and those with HFpEF were not proportionately represented compared to the broader HF demographic, possibly resulting in an underestimation of frailty prevalence among HF patients. The sample exhibited a lack of diversity in terms of race and ethnicity [31]. The effectiveness of ET was again confirmed by a recently published meta-analysis showing decreased hospital admissions for heart failure and other health conditions [58].

##### Interval Training and Continuous Training

An important aspect to consider is the optimal exercise approach for individuals with HF, which includes interval training (IT) and continuous training (CT) [59]. The comparative effectiveness of IT and CT on the cardiac and respiratory fitness and exercise compliance of subjects with HF was assessed througha meta-analysis. The investigation included 17 randomized controlled trials of 617 patients. The results demonstrated that IT led to improvements in oxygen absorption, 6 MWD, and LVET in contrast to CT-treated participants. Nevertheless, on the statistical baseline, no significant changes were observed regarding the respiratory exchange ratio, resting heart rate, or CO_2_ ventilation equivalent slope [37]. The efficacy of high-intensity interval training (HIIT) versus constant training at a modest intensity was tested in another trial involving individuals with HF and coronary artery disease (CAD), specifically measuring exercise ability and various predictive signs. Based on 15 studies with 664 patients, HIIT was found to improve predictors of disease such as LVEF [60].

HIIT shows promise as a superior approach to moderate continuous training (MCT) in reversing heart remodeling and improving the highest levels of VO2 and aerobic capacity, according to limited research. Evidence points to the potential advantages of high-intensity exercise training in improving the physiology, functional status, and quality of life of individuals with heart failure [35]. Following that, it was reported that the inclusion of aerobic training for inspiratory muscles led to a decrease in breathlessness, an increase in maximum VO2, an improvement in quality of life, and an extension of exercise time. A meta-analysis compared inspiratory muscle exercise with a control group and found positive results in subjects with HF, including increased distance traveled in 6 min, highest VO2 value, and breathing per minute. Incorporating exercise to target the inspiratory muscles is now a part of the rehabilitation plan for patients with HF [35].

To establish a hypothetical relationship between aerobic exercise efficacy, quality of life, and respiratory factors, a recent meta-analysis was conducted on subjects with HFpEF and HF. The total number of patients was 399, including 10 studies. HFpEF patients experienced a gradual increase in peak VO2 and health-related quality of life with aerobic exercise compared to the control group. One methodological limitation was combining results from studies on diverse exercise interventions such as aerobic exercise, combined aerobic and resistance training, inspiratory muscle training, and functional electrical stimulation to determine their associations with outcomes, despite the positive results. Another important drawback is the small number of participants in most RCTs. Nonetheless, a thorough exploration of diverse databases with unlimited time constraints and the adoption of particular analysis techniques were implemented to mitigate biases in this study [38].

Two types of research have been carried out to systematically collect evidence, summarize, and validate the efficacy of tai chi in HF treatment [61]. However, additional thorough randomized control trial (RCT) analyses are required to ensure conclusive and reliable outcomes [62].

Another study was performed to explore how traditional Chinese exercise affects HF, indicating it could be a beneficial activity for people with HF [63,64].

Baduanjin, known for over 800 years, is a unique set of fitness exercises that HF patients can try as an alternative exercise [65]. According to traditional Chinese medicine, Baduanjin enhances blood circulation, alleviates breathlessness, and boosts internal organ function through breathing techniques. The training is named “Baduanjin” due to its eight sections with specific movements [66]. Chen et al. discovered that a 12-week Baduanjin intervention had a significant impact on quality of life and physical decline in individuals with heart failure. At weeks 4, 8, and 12 weeks, the control group showed evident positive changes. Despite limited analysis on the effectiveness of Baduanjin in heart failure patients, additional research is needed to explore its impact on quality of life and physical function. Based on this evidence, it will be possible to encourage and practice Baduanjin exercise in a therapeutic setting [39].

#### 2.1.2. Exercise-Based Cardiac Rehabilitation

An important aspect of exercise-based cardiac rehabilitation (ExCR) is its positive impact on symptomatology in patients with HF. Despite strong recommendations for cardiac rehabilitation in HF patients, adherence remains low due to factors like work, service, and patient-related issues [67]. A National Audit of Cardiac Rehabilitation (NACR) UK 2019 report, drawing on evidence from various national studies, highlights the disparity in cardiac rehabilitation participation among different patient groups, particularly women, non-whites, the elderly, ethnic minorities, and those with two or more long-term conditions [68].

A preliminary analysis was performed on data from 13 studies involving 3990 subjects (97% with HFrEF) comparing exercise-based cardiac rehabilitation (ExCR) for a minimum of 3 weeks with a non-exercise control group. The objective of the analysis was to estimate how ExCR affects health-related quality of life, exercise capacity, and differences among HF patient groups. The ExCR showed a significant statistical discrepancy in relation to health-related quality of life and exercise. Conversely, the 6 min walking test and Minnesota Living with HF outcome showed improvements after 12 months. Due to the lack of varying outcomes in cardiac rehabilitation among different patients, researchers propose recommending ExCR for all individuals with HF [69].

The Cardiac Rehabilitation Outcome Study in Heart Failure (CROS-HF) included several randomized trials on exercise-associated cardiac rehabilitation, particularly focused on study groups with more specific left ventricular ejection fraction value < 40%, with a follow-up of 6 or more months. While CROS-HF did not show positive outcomes in terms of patient mortality or hospitalization frequency, it did find that exercise-based cardiac rehabilitation led to improved quality of life and increased exercise capacity [40].

Although cardiac rehabilitation shows promise in terms of results, benefits, and cost-effectiveness, it is still underutilized worldwide, with only 10% to 30% adherence rates. In the HF-ACTION clinical trial, despite making all exercise machines accessible and implementing intense measures to boost compliance, adherence remained below 30% over an extended period. This suggests that there is a growing demand for more accurate patient monitoring and tailored adjustments to enhance adherence, considering the varying symptomatology and tolerance of individuals with HF [35].

### 2.2. HF Nutrition

HF nutrition support involves reducing sodium and liquid intake. Regrettably, individuals with HF, especially those with advanced HF, have a higher likelihood of experiencing various general deteriorations (such as cachexia) or malnutrition [70]. In a Spanish study involving 304 HF patients, lethality in underfed patients (classified using the Mini Nutritional Assessment (MNA) screening test) was higher (68.9%) compared to patients at risk of malnutrition (33.3%) or non-malnourished patients (15.2%; *p* < 0.001) [36].

Based on the information available, patients with HF have lower caloric needs and higher micronutrient deficiencies compared to healthy elderly individuals of the same age and gender [71]. Da Silva Lockmann et al. adjusted the Healthy Eating Index 2015 to better suit the nutritional guidelines for the elderly population. The Healthy Eating Index is an important tool for measuring compliance with food and nutrition guidelines from food guides. This tool is beneficial for overseeing the quality of an individual’s diet in long-term healthcare, measuring the impact of nutritional interventions, and evaluating food and nutrition education initiatives. A version of the Healthy Eating Index was successfully adjusted for the older adult population, demonstrating sufficient validity and reliability. Using the HEI-OA can help nutrition professionals advise older adults on improving their diet quality [71]. Despite the negative impact of reduced vitamin D levels on clinical outcomes in various patient groups, including those with heart failure, its supplementation has not shown clear benefits for clinical performance in subjects with heart failure or endocrine-related diseases. However, nutritional support with hypercaloric, hyperproteic oral supplements, a Mediterranean diet, and vitamin D supplementation were associated with decreased NT-proBNP and improvements in LVEF, functionality, and quality of life in patients with HF, despite a significant decrease in hospital admissions [42,43]. Therefore, further research is required to validate the potential of vitamin D supplementation in treating chronic heart failure patients.

Various theories have been suggested to explain the positive impacts, which are linked to experimental results showing anti-inflammatory, anti-apoptotic, and anti-fibrotic effects, along with shielding against cardiac and subcellular changes. Considering the current pool of unclear evidence, certain discrepancies in the results seem to be linked to factors such as age, gender, race, location, and the metabolic process involved in producing active vitamin D. The effectiveness of vitamin D supplements in treating heart failure is uncertain, but ensuring adequate vitamin D levels via supplementation may help prevent the condition [42,43].

Due to their frailty, elderly HF subjects are reliant on their caregivers for both their diet and overall well-being. The quality of diet in HF subjects and their family support is a crucial problem due to their shared food choices. Using a family frequency questionnaire, a study compared the diet quality of 40 HF patients and their 40 family supporters. Evidence has revealed that patients and family carers derive benefits from lower-quality diets, emphasizing the importance of household-level nutritional interventions [72,73]. Obesity control is another crucial issue in HF [74]. Although obesity is known to increase the risks of HF, there are uncertainties regarding potential adverse outcomes in slim patients with HF. Furthermore, there are limited data on weight loss in individuals with HF who are overweight [75]. Changes in clinical outcomes and adverse events were analyzed in a small study on weight reduction. Despite an acknowledgment of an increase in lifestyle regulation, there has not been a significant decrease in weight. While KSHF and ESC guidelines do not give specific dietary advice, AHA/ACC guidelines for HF recommend following the Dietary Approaches to Stop Hypertension (DASH) and Mediterranean diet, both linked to a reduced risk of HF. An important decrease in HF-related hospitalizations may be linked to the antioxidant and potassium levels in the DASH diet [33].

The latest observation of dietary guidelines for HF, including the DASH diet, was explored in a study with a focus on providing preventive advice for outpatient HF treatment. Subjects diagnosed with HFpEF who followed a low-sodium DASH diet for three weeks observed considerable improvements in ventricular diastolic function and oxidative stress, alongside a notable decrease in hardness and blood pressure. GOURMET-HF’s research reveals that the approval of the DASH diet meal delivery has resulted in symptom reduction, enhanced physical abilities, and fewer hospitalizations among recently hospitalized HF patients [44]. The limited sample size and lack of randomization in interventions restrict the accuracy of DASH nutritional activities in HF trials. In order to determine the true effectiveness of the DASH diet in HF treatment, controlled and randomized clinical trials are needed [45]. The potential of the Mediterranean diet in preventing HF progression has been explored through several studies [76]. The impact of adhering to the Mediterranean diet on clinical results was investigated in a single prospective study of 991 patients with acute heart failure in a Spanish emergency department.

The findings indicated that there was no impact on long-term mortality for patients with acute heart failure, but there was a decrease in hospitalizations over the next year [33].

#### 2.2.1. Prevention of Excessive Salt and Fluid Consumption

A common recommendation for HF patients is to reduce their salt consumption in their diet [77]. To manage moderate-to-severe heart failure, the KSHF advise keeping sodium intake below 2 g per day (equivalent to 7–8 g of salt) [78]. The ESC guidelines advise against consuming excessive salt (>5 g/day) for HF [79]. The AHA/ACC guidelines recommend avoiding excessive sodium consumption for alleviating congestive symptoms in HF patients [33,80].

The idea of limiting salt intake for heart failure patients is controversial [81]. Decreasing sodium intake in the diet of HF patients can be harmful due to the combination of neurohormonal system stimulation and nutritional deficiencies. Conversely, sodium overload in the general balance is primarily caused by fluid retention and volume overload in acute HF. Current research indicates that consuming less salt is associated with a higher risk of readmission and mortality. The question regarding the role of salt restriction in treating HF is still being researched. Personalized nutritional support appears to be more beneficial than standard recommendations, particularly for patients at a greater nutritional risk [82,83]. The highest amount of salt consumption is attributed to modified foods, and European regulations require salt quantity disclosure for each food item, emphasizing personalized decision-making. To prevent the development of cardiovascular disease in hospitals, the ESC 2021 guidelines propose decreasing salt intake by opting for less modified foods and potentially reformulating them to lower salt content [47].

In a recent analysis of 10 trials involving HF patients, dietary sodium restriction (800 mg/day to 3000 mg/day) did not improve the quality of life. Long-term dietary sodium restrictions did not have an impact on the frequency of hospitalization or mortality in patients with HF [40].

SODIUM-HF (Study of Dietary Intervention under 100 mmol in Heart Failure Trial) was recently published. Its purpose was to assess the impact of limiting salt intake to less than 1500 mg per day on clinical outcomes in HF patients. This trial is open to participants worldwide and involves randomization and control for individuals with chronic heart failure, following clinical guidelines for optimal medical therapy. A total of 806 subjects were included in the study, with random selection for either regionally prescribed standard therapy or a low-sodium diet. In the low-sodium diet group, 15% of participants experienced hospitalization or death due to cardiovascular reasons or other causes compared to 17% in the conventional treatment group (hazard ratio 0.89). There were no safety events reported in either group that were linked to the study therapy. According to the study, reducing dietary salt intake (up to 1500 mg/day for HF patients) did not show any improvement in clinical events, such as deaths, hospitalizations, or cardiovascular-related ER admissions, compared to standard therapy for one year. While New York Heart Association (NYHA) function and patient-reported quality of life saw improvements, there were no significant distinctions in the group’s 6 min walking distance. In order to determine the impact of reducing dietary salt on clinical outcomes, patients and experts should examine the efficacy of dietary intervention compared to other medical therapies, taking into account individual benefits [46]. Several key limitations are associated with this study. The lack of masking for patients regarding their study group assignment, in contrast to the blinded intervention and outcome assessment, could have introduced bias, particularly affecting secondary outcomes such as NYHA functional class, KCCQ, and 6 min walking distance. Nonetheless, the food records gathered from both study groups indicated a decline in dietary sodium without notable variations in other diet-related factors (like calorie or fluid consumption), making it doubtful that this is a major bias factor. In addition, events were centrally reviewed in a blinded manner to decrease bias related to treatment assignment. Moreover, it is possible that some patients in the usual-care group decided to reduce their sodium intake (contamination bias) [46].

Unlike sodium restriction, the potential therapeutic advantages of salt consumption have been examined for HF treatment. A thorough analysis was conducted to understand how sodium control is achieved in euvolemic patients with HFrEF after following recommended medical treatment. Participants in the same age group were included in the research after consuming a slightly higher sodium intake of 1.2 g (51 mmol) daily for 4 weeks. Patients who had proper support for heart failure and reduced ejection fraction were able to handle an ongoing increase in sodium intake without any manifestation of heart failure symptoms or signs. Furthermore, the rise in blood volume and sodium intake caused a significant reduction in neurohormonal activation and an increase in sodium excretion [47].

Even though it is generally recommended, clinical guidelines do not provide precise indications for fluid restriction [78]. Fluid restriction should be implemented for HF patients at the time of hospital discharge, as suggested by the KSHF guidelines [79]. ESC guidelines recommend limiting fluid intake to 1.5–2 L for severe HF patients with hyponatremia to alleviate symptoms and congestion [32]. The AHA/ACC guidelines indicate that there is likely uncertainty regarding the advantages of fluid restriction for patients with advanced HF and hyponatremia. However, the basis for these suggestions is extremely limited in terms of evidence [33,39]. Research conducted by Travers et al. demonstrated that subjects with acute heart failure, regardless of their fluid intake, had no significant variations in the time required to attain clinical stability. Nevertheless, the difference in fluid consumption between the groups was only 392 mL/day. The Strict Allowance of Fluid Therapy in Hyponatremic Heart Failure (SALT-HF) pilot study suggests that adopting a low-fluid diet could enhance the quality of life for patients with low blood sodium levels (<135 mmol/L). Unluckily, thirst can continue to be a long-standing issue for around 50% of heart failure patients, particularly when hyponatremia is present, greatly affecting their quality of life as they have to restrict fluid intake [47].

Findings from a recent experimental trial revealed that subjects who received a behavioral and educational intervention, resulting in a slight reduction in fluid consumption, reported decreased HF symptoms, heightened thirst sensation, and maintained health-related quality of life [80].

The possibility of a correlation between chronic dehydration and HF was investigated by Dmitrieva et al. using data from the Atherosclerosis Risk in Communities (ARIC) study, a research study in the United States (US) that followed a diverse cohort of 15,792 individuals for more than 25 years. Serum sodium levels of the study subjects were used by the authors as an indicator of hydration during two visits spaced three years apart. In the study, 11,814 participants with HF were selected who had regular water status, normal blood sodium levels, were not overweight, and were not diabetic. In middle-aged subjects, blood sodium levels over 143 mmol/L corresponded to a 39% greater risk of developing heart failure, according to the analysis [48].

Hence, more randomized trials are needed to establish the safety and functionality of fluid restriction. The main focus of the FRESH-UP study was to suggest fluid restriction for HF patients, but there is a lack of clinical trial results. As previously stated, limiting fluid intake can enhance the feeling of thirst and have a negative effect on one’s quality of life. The FRESH-UP research project aimed to tackle this deficiency by studying fluid restriction in heart failure vs. liberal fluid uptake. The methods encompass a clinical trial that is open-label, randomized, controlled, and multicenter. The consequences of fluid restriction (1500 mL/day) will be analyzed in comparison to unrestricted fluid use for three months. This study focuses on the quality of life of hospitalized patients with chronic HF. The main goal is to assess the impact on quality of life after three months through the Kansas City Cardiomyopathy Questionnaire (KCCQ) overall summary score. Other outcomes encompass patient-reported fluid consumption, the clinical summary score of KCCQ, safety measures (deaths, HF hospitalizations), each KCCQ category, and substantial growth in these scores [84].

In dehydration, stress plays a crucial role by limiting fluid intake in humans, regardless of its cause or association with physiological processes. Typically, water diffuses from the intracellular to the extracellular compartment in order to keep blood pressure and plasma volume in check during stressful situations. Multiple hormonal mechanisms (renin–angiotensin–aldosterone system (RAAS), antidiuretic hormone (ADH), atrial natriuretic peptide (ANP), and cortisol) control water levels to prevent negative effects [28].

#### 2.2.2. Body Weight Management

Guidelines recommend weight loss to prevent the progression of heart failure, but its effectiveness in treating advanced heart failure is still uncertain [39,82].

Although obesity is associated with a higher likelihood of heart failure, it paradoxically provides protection in diagnosed patients, as a lower body mass index (BMI) is connected to increased mortality risk. HF has been detected independently and unaffected by other medical conditions or the type of HF [83,85].

The “obesity paradox” has raised concerns among researchers about the impact of weight loss on the prognosis of heart failure patients and is mainly observed in heart failure patients with a BMI between 30 and 34.9 kg/m^2^, but not in those with a BMI of 35 kg/m^2^ or higher. To examine the effects of an intensive lifestyle change program on weight loss and functional status improvement, a short study was carried out on 41 patients with HFpEF and obesity who had an average BMI of 40.8 kg/m^2^. After 15 weeks, the mean 6 min walking distance increased to 281 m (*p* = 0.001), but later decreased to 267 m after 26 weeks. The Minnesota Living with Heart Failure (MLHFQ) score saw a decrease from 59.9 to 37.3 after 15 weeks (*p* = 0.001), and remained stable at 37.06 after 26 weeks. Changes in body weight were associated with changes in both MLHFQ score and the 6 min walking distance [86].

In terms of weight loss, research by Mahajan et al. revealed that bariatric surgery has a significant effect on improving heart function and structure. The effects of weight reduction were examined in a Swedish registry survey of 40,000 individuals without heart failure through intensive lifestyle interventions or bariatric surgery. The cohorts had similar baseline weight and BMI values. Surgery led to significantly greater weight loss compared to lifestyle interventions, with a loss of 18.8 kg more after one year and 22.6 kg more after two years of follow-up. The rate of heart failure development was lower with surgery compared to lifestyle change after a 4.1-year follow-up (4.1% vs. 7.6% per 10,000 person-years) [50].

Observations show that chronic stress can affect body areas with stress hormone receptors. The assessment of functional body structure can provide important information about individuals’ general well-being, taking into account the changes in body composition and chronic stress. To ensure individual well-being, it is necessary to screen individuals using advanced medical techniques such as bioelectrical impedance analysis (BIA) and dual-energy X-ray absorptiometry (DXA), as there is a significant association between body changes and stress. This allows for timely prevention or treatment.

The improvement in sleep apnea in multiple patients can be supported by heart failure therapy and reducing body weight. Nonetheless, the outcomes remain uncertain [87].

An efficacy trial of adaptive servo-ventilation (ASV) therapy in chronic HF patients revealed unfavorable outcomes for left ventricular ejection time (LVET) and plasma brain natriuretic peptide (BNP) concentrations during a 24-week period. Despite this, the therapy was still able to improve patients’ quality of life and clinical status [51].

Continuous positive airway pressure (CPAP) has been found to provide several advantages in HFrEF through randomized clinical trials, such as improvements in apnea and hypopnea, nighttime awakenings, daytime systolic pressure, heart rate, LVEF increase, and reduction in left ventricular end-diastolic diameter (LVEDD). CPAP treatment has significantly reduced mortality in HFrEF patients, particularly in those who received treatment [52].

The results of a randomized controlled clinical trial revealed that patients with obstructive sleep apnea (OSA) and heart failure experienced improved LVEF and a decrease in urinary norepinephrine elimination. Colish showed that there was a higher rate of hospitalization or death in non-CPAP subjects with heart failure and moderate-to-intense OSA compared with patients who received CPAP treatment. Initially, bias and diversity were observed in the acupuncture treatment for OSA. The various approaches of acupuncture could be a reason for this. Furthermore, patients with OSA were categorized as mild, moderate, or severe using their apnea–hypopnea index (AHI) scores before treatments. The AHI proves to be a helpful resource for diagnosing and arranging the severity of OSA patients’ conditions. Nevertheless, there are inherent limitations in using the AHI determined from a single night of sleep to gauge the severity of the disease. Various factors can influence the AHI, resulting in changes over time and between nights. In general, the methodological quality of the RCTs included was considered to be low. A large number of the studies reviewed exhibited a high likelihood of performance bias [88].

### 2.3. The Link Between Addiction and HF Management

Substance abuse is common in individuals with heart failure and is linked to unfavorable clinical outcomes [89]. The use of drugs such as cocaine, cannabis, smoking, and alcohol can be indicative of the development of heart failure [90]. While moderate alcohol consumption may slow HF advancement, excessive use can lead to dilated cardiomyopathy. According to a prospective trial in 2018 that lasted 82 months, it was established that reducing or ceasing alcohol consumption can promote the appropriate recovery of left ventricular ejection fraction (LVEF) [51].

Substance abuse disorders have significant health implications and are causes of death, but they are not directly associated with heart failure hospitalizations or degeneration, despite being less prevalent than other heart failure-related conditions. Improved identification and treatment of medication abuse could be helpful for individuals with HF [91]. The specific amounts of alcohol that lead to disease development are unclear, although we currently understand the epidemiological mechanisms related to HF and cardiomyopathy in alcohol abusers [51].

Regardless, patients should be open to suggestions on preventing ongoing alcohol consumption. It is imperative to reduce or eliminate alcohol abuse as a key objective in the treatment of patients with alcoholic cardiomyopathy. According to a meta-analysis of 84 trials, consuming 2.5–14.9 g/day of alcohol reduced the risk of death from cardiovascular causes, coronary heart disease, and stroke development by 14–25% [54]. Tobacco use is linked to an increased risk of heart failure, both through (CAD) and other mechanisms unrelated to CAD. Smoking negatively affects individuals with HF, and death is associated with a decrease in serious adverse cardiac effects. Smoking patients with heart failure require special care, as even a minor reduction in smoking can have therapeutic benefits for HF patients. The risk of smoking-related heart failure persists in older patients regardless of their smoking history, and cigarette smoking correlates with adverse changes in blood biomarkers of HF pathophysiology [92].

Further investigation is crucial for a better comprehension of the impact of these drugs on the development of heart failure and their potential effects on individuals already suffering from HF [89].

## 3. Nutritional Supplementation in Prevention and Therapy Support of HF

Extensive research shows that a deficiency in minerals and vitamins is associated with a higher risk of HF, and a specific nutritional supplementation can enhance HF-related factors, even alongside conventional treatments. (Table 3) [93]. HF development and progression are significantly impacted by nutrition. Particularly in the early stages of HF, it is viable to identify dietary causes of its progression, which include a surplus of calories, excessive consumption of processed foods high in salt and calories, and insufficient consumption of legumes, nuts, fruits, and vegetables [94]. The presence of vitamin C in fruits and vegetables is connected to a decreased HF risk. A few studies have explored the relationship between plasma vitamin C levels and HF incidence, indicating a positive association between higher plasma vitamin C levels and lower risk of HF [95]. Polyunsaturated fatty acid (PUFA) supplementation has the potential to yield positive outcomes for HF patients, particularly in the early stages, as supported by some evidence. Docosahexaenoic acid (DHA), when taken at doses relevant to clinical settings, has the ability to enter mitochondrial membranes and mitigate the risk of mitochondrial permeability transitions triggered by Ca^2+^ and stress [93]. On the other hand, ferric carboxymaltose (FCM) supplementation showed a significant improvement in cardiac function in patients with HF and iron deficiency [96]. Additionally, there is evidence to support the potential activity of CoQ10 treatment to ameliorate the symptoms of HF patients [97].

### 3.1. Supplementation with Ferric Carboxymaltose (FCM)

#### 3.1.1. Iron Deficiency and HF

Iron deficiency (ID) with or without associated anemia is a factor that has not been fully explored yet in the context of heart failure and in identifying effective treatment for HFpEF [115]. The presence of anemia is a common finding in patients with HF, with a prevalence ranging from 4% to 61%. This variability could be due to the heterogeneity of HF and the spectrum of disease severity in different studies [116]. HF was associated with a higher prevalence of ID in studies of advanced forms of reduced myocardial performance and lower functional capacity. Anemia can occur in HF patients as a result of different etiologies, including a reduced erythropoietin response [117]. ID in HF can occur due to appetite loss, inadequate nutrition, reduced iron absorption from edema, and gastrointestinal bleeding caused by anti-platelets and anticoagulants [118]. Iron-limited erythropoiesis, which is known as functional iron deficiency, is a form of anemia caused by chronic inflammation controlled by hepcidin, a peptide that inhibits intestinal iron absorption and iron release from circulating macrophages. In HF, inflammation can trigger hepcidin expression, resulting in inadequate iron absorption. The presence of anemia and HF is a proven predictor of morbidity and mortality [119]. Specifically, iron deficiency is a powerful independent predictor of negative outcomes in systolic HF. In a prospective observational study of 546 patients with stable systolic HF, ID with or without anemia was an independent predictor of death or heart transplantation [115]. ID can result in reduced aerobic performance and the development of exercise failure. In a single-center cross-sectional study of 538 stable patients with chronic heart failure, iron deficiency had an independent and linear association with submaximal exercise capacity based on the 6 min walking test (6 MWD) and with symptomatic functional limitation based on NYHA class of reference [120]. Increased hospitalization has been linked to the presence of ID in HF, with significant impairment in health-related QoL [121].

#### 3.1.2. IV Iron Treatment in HF

Bolger conducted a single-arm study in 2006, examining IV iron treatment in 16 patients with heart failure and systolic HF. The patients received 1 g iron sucrose daily for 12 days, targeting those with inappropriate iron deficiency and ferritin levels of 400 ng/mL. An improvement in the 6 MWD test and MLHFQ was noted [98]. In 2007, Toblli et al. conducted a randomized study on 40 HF patients with an ejection fraction (EF) of 40%. They were divided into two groups, one receiving normal saline and the other receiving intravenous (IV) injections of 200 mg sucrose per week for 5 weeks. The inclusion criteria encompassed anemia and a hemoglobin (Hb) concentration of 12.5 g/dL in men and 11.5 g/dL in women, in addition to iron deficiency defined by serum ferritin levels of 100 ng/mL and/or transferrin saturation (TSAT) of 20%. Patients who were given IV iron exhibited lower levels of N-terminal pro-B-type natriuretic peptide (NT-pro-BNP), improved functional capacity, and a reduced number of hospitalizations over the course of 6 months [99]. In 2008, Okonko et al. carried out a randomized study of 35 patients with an EF of 45%, anemia (defined as Hb concentration 12.5 g/dL), and evidence of ID (defined as ferritin 100 ng/mL or ferritin between 100 and 300 ng/mL and TSAT 20%), who were administered 1400 mg of iron sucrose or saline. Although there were no significant differences in peak oxygen consumption or treadmill exercise duration (*p* = 0.08 for both), significant improvements were observed in NYHA functional class and global patient assessment [100]. Due to the small sample, it was challenging to detect slight differences between the treatment groups. Despite the risk of subject and/or investigator bias, the primary endpoint was objectively determined, impervious to bias, and blindly assessed. The ferritin level indicating iron deficiency was above the threshold set by the World Health Organization. Elevated ferritin cutoffs ensure high sensitivity and specificity in identifying iron deficiency in patients with chronic heart failure and inflammation [100].

#### 3.1.3. IV FCM Treatment in HF

Ferric carboxymaltose (FCM) is a macromolecular complex of ferric hydroxide and carbohydrate that allows for controlled delivery of iron within the reticuloendothelial system and the transport of the iron-binding proteins ferritin and transferrin, with no chance of excessive ionic iron release in the serum [122]. The supplementation of FCM in patients with HFrEF and iron deficiency reduced symptoms and improved quality of life [123]. The presence of anemia resulted in a greater observed improvement. The most significant advantage of intravenous iron in heart failure patients was seen in the FAIR-HF study. The study enrolled patients who did not have anemia in another cohort study. Patients were randomly assigned in a 2:1 ratio receiving 200 mg IV of FCM or saline. The protocol included a maintenance dose every 4 weeks if serum ferritin, Hb, or TSAT fell below predefined values. Endpoints were evaluated at 24 weeks. According to the overall patient assessment, 50% versus 28% of patients showed improvement with FCM (odds ratio [OR] for improvement, 2.51; 95% CI 1.75–3.61). In the group assigned to FCM, 47% were in NYHA functional class I or II at week 24 compared to 30% assigned to placebo (OR for improvement of one class, 2.40; 95% CI 1.55–3.71). In the group assigned to FCM, 47% were in NYHA functional class I or II at week 24 compared to 30% assigned to placebo (OR per class improvement, 2.40; 95% CI 1.55–3.71). Results were consistent regardless of anemia status [101]. Significant improvements were also reported in the treatment arm in the 6 MWD and QoL questionnaire. The difference in serum ferritin levels was 246 ng/mL and the difference in Hb concentration was 0.5 g/dL, mainly due to the difference in anemic patients (0.9 g/dL in anemic patients compared to 0.1 g/dL in those without anemia). A placebo-controlled study yielded an almost normal Hb concentration (12.5 g/dL at the end of the trial). It can be concluded that there were benefits of FCM even in the absence of documented previous anemia. The rates of death or adverse events in both study groups were comparable. In total consistency with the FAIR study were the data from the CONFIRM-HF study published in 2015, which was a multicenter placebo-controlled study that enrolled 304 symptomatic outpatients with HF with left ventricular EF ≤ 45% and iron deficiency randomized 1:1 to treatment with FCM or saline and followed up for 52 weeks [102]. This study had the longest published follow-up period in patients with heart failure treated with iron. Treatment with FCM significantly improved the 6 MWD test at week 24 (FCM vs. placebo difference: 33 + 11 min; *p* = 0.002), and this treatment effect of FCM was consistent across subgroups and remained constant until week 52 (FCM vs. placebo difference: 36 + 11 min; *p* = 0.001) [102]. In addition, improvements in NYHA class and other functional/QoL measures with statistical significance were reported from week 24 onwards. Furthermore, treatment with FCM was associated with a significant reduction in the risk of hospitalization for worsening HF (HR 0.39, CI 95% 0.19–0.82; *p* = 0.009). Mortality and the incidence of adverse events were similar between the study groups [102]. Although the combined evidence from the abovementioned studies demonstrated an improvement in functional capacity and QoL after IV, this therapy only partially translated into a reduction in HF hospitalizations and did not result in a survival benefit [103]. The impact of intravenous supplementation FCM on 64 subjects with hypotrophic erectile dysfunction (HFE) was examined in a blinded, randomized, placebo-controlled study conducted simultaneously in multiple centers. Ferritin level measurements were used to identify ID and correlate with cardiac performance measurements from a six-minute walking test and echocardiographic measurements of distal function. Moreover, an EndoPAT analysis was performed to link cardiac function to endothelial dysfunction, and malondialdehyde (MDA) levels in serum were assessed to uncover biomarkers of oxidative stress. Measurements were performed before and 8 weeks after starting treatment with either a placebo (100 mL of saline delivered by injection in 10 min; n = 32) or FCM at a dose of 500 mg IV infusion (n = 32), which was administered at the beginning and repeated four weeks later. The study highlighted that ID had a higher incidence of impaired diastolic function, worse 6 MWT, and endothelial dysfunction, an outcome that was associated with high MDA serum levels [96].

### 3.2. CoQ10 Supplementation

#### 3.2.1. Coenzyme Q10 Activity

Coenzyme Q10 (CoQ10), also known as ubiquinone, is a lipid-soluble cofactor involved in the mitochondrial electron transport chain. It can be synthesized internally or obtained from the diet, and its role is to transfer electrons between complexes I and II to complex III. The transfer of protons and electrons by CoQ10 leads to the creation of a transmembrane potential, which adenosine triphosphate synthase (ATP) utilizes to produce ATP [124]. CoQ10 acts as a potent antioxidant in biological membranes, such as mitochondria, by preventing lipid peroxidation and supporting cellular maintenance [125]. Lipoproteins play a role in transporting CoQ10, and its plasma levels are connected to plasma total low-density lipoprotein (LDL) cholesterol [126]. Tissues that require a lot of energy, like the heart, skeletal muscle, and neurons, have high concentrations of CoQ10. While aging and oxidative stress lead to a decrease in its tissue concentration, its bioenergetic role becomes more prominent.

#### 3.2.2. Coenzyme Q10 and Cardiovascular Disease

**Figure 2:**
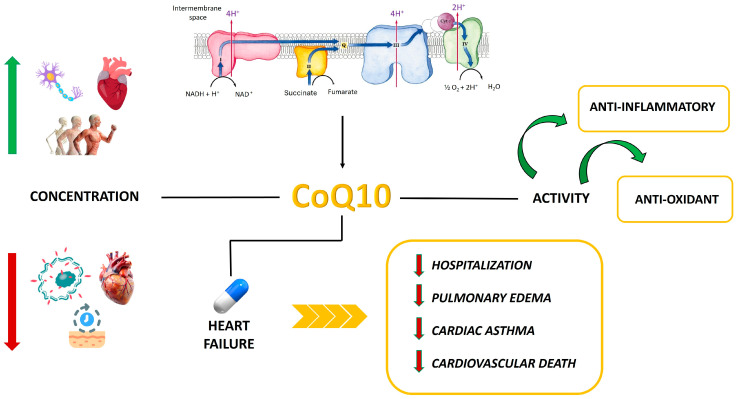
Coenzyme Q10 (CoQ10) is a fat-soluble cofactor that plays a role in the mitochondrial chain of electronic transport. In biological membranes, including mitochondria, CoQ10 acts as a strong antioxidant by blocking lipid peroxidation and has important anti-inflammatory properties, protecting myocardial tissue. Energy-intensive tissues, such as the heart, skeletal muscle, and neurons, have high concentrations of CoQ10, while aging and oxidative stress lead to a decrease in its concentration. Long-term administration of CoQ10 in addition to standard therapy for chronic HF led to a lowering in hospitalizations for heart failure, episodes of pulmonary edema, and cardiac asthma, reducing cardiovascular death.

Additionally, patients with heart failure witness a decrease in CoQ10 levels within their heart muscle, with the deficiency intensifying as heart damage worsens, highlighting its significance in preventing cardiovascular disease [127]. By exerting antioxidant effects, CoQ10 can potentially decrease oxidative stress, which can negatively impact left ventricular ejection fraction and affect disease outcome. In a study, 70 patients who had their first ST-elevation myocardial infarction (STEMI) and qualified for primary percutaneous coronary intervention (PPCI) were randomly assigned to one of two groups. The first group received standard treatments along with CoQ10 (400 mg before PPCI and 200 mg twice daily for three days after PPCI), while the second group received standard treatments along with a placebo. Both groups exhibited similar plasma levels of oxidative stress biomarkers, including superoxide dismutase (SOD), catalase (CAT), glutathione peroxidase (GPx), total antioxidant capacity (TAC), and MDA, at 6 and 24 h. Conversely, by the 72 h mark, the CoQ10-treated group demonstrated notable improvements. Additionally, they exhibited significantly elevated plasma levels of SOD and CAT, along with a significant reduction in MDA. It is suggested by the results that CoQ10’s antioxidant capacity may aid in reducing reperfusion injury in the ischemic myocardium [110]. Besides its antioxidant and bioenergetic function, CoQ10 has been shown in preclinical studies to possess significant anti-inflammatory properties, safeguarding myocardial tissue from fibrosis and alterations in left ventricular structure via NO regulation. Indeed, CoQ10 demonstrated a reduction in reactive oxygen species generation and an improvement in antioxidant capacity in endothelial cells (HUVECs) exposed to oxidized low-density lipoprotein (oxLDL). Additionally, CoQ10 prevented the oxLDL-mediated suppression of endothelial nitric oxide synthase (eNOS) and the induction of inducible nitric oxide synthase (iNOS). Moreover, CoQ10 suppressed the activity of nuclear factor kappa-light-chain-enhancer of activated B cells (NF-κB) and downstream inflammatory mediators, which included adhesion molecule expression, proinflammatory cytokine release, and adherence of monocytic THP-1 cells, attenuating the pro-apoptotic signals induced by oxLDL [128]. Statins are prescribed for HF patients as they inhibit cholesterol synthesis, but they also block the mevalonate pathway, which reduces endogenous CoQ10 production, leading to decreased blood CoQ10 levels during statin therapy [129]. The Controlled Rosuvastatin Multinational Study in HF (CORONA) revealed that patients with low CoQ10 levels had higher mortality, lower LVEFs, and increased levels of NT-proBNP. Despite reducing CoQ10, rosuvastatin treatment did not significantly worsen outcomes, even in patients with low baseline CoQ10. Instead of predicting, it is suggested that CoQ10 levels may reflect the severity of the disease [130]. In the 1960s, groundbreaking research began on the potential advantages of CoQ10 in enhancing heart function for adults with HF. Increasing evidence has supported the use of CoQ10 as an adjunct to conventional therapy for HF in adults [131]. The focus of these trials has been mainly on patients with HFrEF until now. In a large, randomized trial completed in 1993, CoQ10 was found to decrease the risk of HF hospitalization and episodes of pulmonary edema and cardiac asthma compared to a placebo group. In the group treated with coenzyme Q10, there were fewer patients requiring hospitalization for worsening heart failure and a lower incidence of serious complications in patients with chronic congestive heart failure compared to the control group [111]. According to the Q-SYMBIO trial, which involved 420 participants and ended in 2014, the long-term administration of CoQ10, along with standard therapy for chronic HF, led to a decrease in the primary 2-year endpoint. The endpoints encompassed cardiovascular death, hospitalizations for heart failure, mechanical support or heart transplant, death from cardiovascular causes, and all-cause mortality [112]. The lack of patentability for CoQ10 and the low budget of Q-SYMBIO made it hard to compete with other HF trials that used licensed pharmaceuticals. This clarifies why the study did not follow the initial enrollment plan and why the expected 550 patient count was not achieved. Approximately 20% of patients in both treatment groups were already on standard therapy without diuretics when the study started. The death rate after 2 years being lower than anticipated might be because more patients had milder symptoms [112]. In a European subpopulation, a subgroup analysis of the Q-SYMBIO trial confirmed that CoQ10 is therapeutically effective. It reduced major adverse cardiovascular events, hospitalizations, and cardiovascular mortality, while also improving symptoms. After two years, the CoQ10 group had lower all-cause mortality (53%) compared to placebo. Hospitalization due to worsening HF significantly decreased in the CoQ10 group (3%) compared to the placebo group (13%) [113]. Myocardial bioenergetics undergo changes in diastolic dysfunction, which is the main feature of HFpEF. Furthermore, inflammation and increased reactive oxygen species (ROS) production may lead to endothelial dysfunction, causing harmful cardiac remodeling and subsequent impairment of ventricular relaxation. The understanding of CoQ10’s role in mitochondrial bioenergetics has justified the study of its potential as a treatment for HFpEF (Figure 2) [132,133].

There is a lack of investigation into the relationship between CoQ10 and left ventricular diastolic function (LV) in HFpEF patients in previous studies. The only trial conducted was a pilot, randomized, double-blind, placebo-controlled study in HFpEF patients over 55 years old, held from June 2017 to December 2019. Serum NT-proBNP levels and echocardiography were assessed before and after a 4-month trial of CoQ10 or placebo, but no clinical benefit was observed from CoQ10 supplementation. The absence of results might be due to the fact that HFpEF exhibits different clinical phenotypes and responds differently to various therapeutic strategies [114]. Identifying a specific group of HFpEF patients who respond positively to CoQ10 treatment may be possible through the targeted assessment of myocardial energetics [114].

### 3.3. Polyunsaturated Fatty Acid (PUFA) Supplementation in Patients Suffering from HF

Polyunsaturated fatty acids (PUFAs) play a key role in several physiological processes and are involved in the regulation of inflammation, vascular hemodynamics, antioxidant protection, and other vital biological functions. A significant clinical classification is the distinction between n-3 PUFAs with the end double bond at C3, starting from the methyl end of the hydrocarbon chain, and n-6 PUFAs with the end double bond at C6 [134]. Omega-3 (Ω-3) and omega-6 (Ω-6) PUFAs are considered “essential” because they cannot be produced by the body, but need to be assimilated with the diet [135]. In a meta-analysis study involving nearly 80,000 heart failure patients, it was determined that Ω-3 fatty acids did not have a substantial impact on heart failure admissions or cardiovascular mortality, but did have a slight but significant effect on rehospitalizations. A total of 81,364 patients were included in the study, with follow-up times spanning from 6 months to 6.2 years. The average (standard deviation) age of the group was 65.3 (8.7). In 1988 cases, there was no significant difference in the rate of first hospitalization for HF between the randomized groups. Furthermore, the randomized groups did not show a statistically significant difference in cardiovascular mortality. Ω-3 treatment resulted in a significantly lower hospitalization rate for recurrent HF compared to placebo treatment [104]. One significant and frequent restriction in current trials is that HF was not predetermined as the primary outcome, and the limited statistical power in each trial could have contributed to the variation in HF results [104]. A comprehensive review analyzed the most significant clinical studies on the effect of PUFA supplementation in HF. The Multi-Ethnic Study of Atherosclerosis (MESA) was the biggest clinical study ever conducted to assess the role of omega-3 fatty acids in preventing the occurrence of heart failure. Approximately 6562 participants (52 females) were part of the MESA cohort, which included Asians, US whites, Hispanics and African-Americans aged 45–84 years. The average duration of follow-up was around 13 years. The plasma concentration of eicosapentaenoic acid (EPA) was measured and expressed as %EPA. According to Block R.C. et al., a higher plasma %EPA correlates with decreased risk of HF, specifically HFrEF and HFpEF, which is also valid for the other Ω-3, particularly for the %EPA + %DHA combination [105]. Additionally, some studies demonstrated the beneficial impact of n-3 PUFA treatment on heart rate and BNP levels in patients with HFrEF, reducing ventricular wall tension. This evidence supports the use of Ω-3 in reducing death and rehospitalization in HFrEF patients. Because of the common association between obesity and HFpEF, clinical studies have been carried out in obese patients with HFpEF. Evidence from clinical trials suggests that consuming a diet rich in UFAs (monounsaturated fatty acids (MUFAs) and PUFAs) improves diastolic function and cardiorespiratory efficiency, leading to increased fat mass [106]. The primary limitation is the poor quality of some studies that were included, with information lacking on allocation concealment or blinding. Furthermore, a number of studies have experienced significant dropout rates, primarily attributed to the duration of the trials. Moreover, despite our use of the tool to review the studies, the assessment of bias is a subjective matter. No specific measure exists to assess artificial bias risk. Thirdly, the limited number of studies included was due to our strict criteria for inclusion and exclusion, possibly affecting the evidence’s robustness. Nonetheless, an innovative drug with the potential to alter the course of heart failure, an angiotensin–neprilysin inhibitor (LCZ696), demonstrated a substantial 20% decrease in the risk of cardiovascular death and heart failure readmission, along with an approximate 20% reduction in total mortality [106].

A retrospective cohort study conducted by Matsuo N et al. enrolled 140 patients suffering from acute decompensated HFpEF. It was found that patients with low docosahexaenoic acid (DHA) levels were at higher risk of all-cause death based on the assessment of plasma levels of EPA, DHA, arachidonic acid (AA), and dihomo-γ-linolenic acid (DGLA). Utilizing DHA plasma levels could be beneficial as both a diagnostic tool and a target for therapies in patients. The EPA/AA ratio was studied in 577 HF patients divided into two groups, and its correlation with HF patient mortality was discovered. The first group consisted of EPA/AA ratios below 0.32 mg/dL, while the other group had EPA/AA ratios of 0.32 mg/dL or higher. No differences were found in parameters such as BNP levels, blood pressure, or LVEF. On the other hand, variations in cardiac mortality rates exist, and they are inversely related to the EPA/AA ratio. This indicates that the ratio can independently predict cardiac mortality in patients with HF. The evidence indicates a strong correlation between this result and statin use in HF patients [107]. Following the screening of 1132 studies, 12 RCTs were incorporated into this study. In total, 81,364 patients were analyzed, and their follow-up periods varied from 6 months to 6.2 years. The average age of the patients involved was 65.3 (8.7) years in 1988 cases. The randomized groups had no significant differences in the rate of first HF hospitalization, which occurred in 1988 cases, with 1011 receiving placebo and 977 Ω-3. Additionally, the placebo groups showed no significant disparity in cardiovascular mortality, with 990 cases for Ω-3 and 948 cases for the control group. Compared to placebo, Ω-3 patients experienced a significantly decreased rate of recurrent HF hospitalization [108]. In this research, the serum level of alpha-linolenic acid (ALA) in patients with HF was investigated. A median follow-up of 2.4 years (0.02–3 years), revealed 85 cardiovascular deaths, 140 all-cause deaths, and 141 first HF hospitalizations (which included all-cause of death and first HF hospitalization, n = 238) were documented. The risk of composite deaths and first HF decreased significantly for patients in the three upper quartiles (Q2–Q4) compared to those in the lowest quartile of ALA in serum (Q1) using cyclooxygenase enzyme (COX) regression analyses (HR: 0.61; 95% CI: 0.46–0.81). The decrease in all-cause death was statistically significant (HR: 0.58; 95% CI: 0.41–0.82), as for cardiovascular death (HR: 0.51; 95% CI: 0.32–0.80), first HF hospitalization (HR: 0.58; 95% CI: 0.40–0.84), and the composite of cardiovascular death and HF hospitalization (HR: 0.58; 95% CI: 0.42–0.79). During the midterm follow-up, patients with the highest ALA levels had a better prognosis than those with lower levels. To enhance quality of life, dietary interventions rich in ALA could be tested on this population [109]. Increasing levels, whether through diet or medication, have been reported to improve outcomes in the heart failure population. Observational studies and intervention trials have limitations due to heterogeneity within the heart failure population, so caution must be exercised when interpreting these findings. Additionally, variations in study design, including dosing of Ω-3, and a lack of data on people with HFpEF contribute to the overall limitations [136].

## 4. Recent Preclinical and Clinical Evidence in Nutraceutical Supplementation and HF

Nutraceuticals have been under investigation for their potential in treating HF-related symptoms for the past 30–40 years. According to available data, including studies and trials, nutraceutical supplementation has been found to improve various parameters related to heart failure, even when combined with conventional treatment [93]. The heart-protective benefits of nutraceuticals might be attributed to their anti-inflammatory, antioxidant, lipid-lowering, anti-ischemic, and angiogenic qualities [137]. Clinical outcomes have shown the potential of nutraceuticals in preventing HF and treating early pathology, either on their own or when used alongside drug therapy [138]. The intake of specific nutraceuticals has been linked to improvements in functional parameters like stroke, ejection fraction, volume, and cardiac output in HF patients, with minimal side effects reported in clinical studies. Further meta-analyses supported these findings, suggesting that the benefits were more pronounced during the early stages of HF. The main mechanisms involved are anti-ischemic, anti-inflammatory, antioxidant, and anti-platelet effects [137,138]. Several plant extracts, including Malus domestica derivatives, Vitis vinifera seed extracts, Citrus Bergamia polyphenolic fraction and Olea europaea L. extracts, showed a significant improvement in endothelial dysfunction, oxidative stress, inflammation, and glycemic and lipid profile, which are the principal features of metabolic and cardiac alterations that can lead to congestive HF (Table 4) [137,139]. Since the current therapies to improve or prevent cardiovascular damage show a narrow field of action, the combination of nutraceuticals could promote mechanisms to repair the damage underlying the onset or progression of the pathology. Therefore, in the following paragraphs, we will examine the main mechanisms and the preclinical and clinical results of some of the major nutraceuticals that have yielded promising results for evaluation in adjuvant therapy of HF. While preliminary approaches indicate the usefulness of nutraceutical supplementation in early-stage HF patients, recent studies and meta-analyses highlight the insufficiency of evidence to support their widespread use in HF patients [23]. Due to the few studies performed and the inconsistent number of study participants, there are major weaknesses in the existing evidence, which results in decreased study quality and high risk of bias. As a result, clinicians tend to prioritize other treatments that have demonstrated a decrease in mortality, regardless of the lack of definite conclusions [24]. Hence, while nutraceutical supplementation shows promise in preventing and treating cardiovascular disease, its potential for treating HF still requires thorough evaluation [25]. Additional studies are needed to determine the most effective usage of nutraceutical supplementation in treating myocardial dysfunction in HF patients.

### 4.1. Malus Domestica Derivatives

Phlorizin, the primary phenolic glycoside found in apple trees, is a competitive inhibitor of sodium–glucose cotransporter 2 (SGLT2) and has shown potential effectiveness [163,164,165].

The use of phlorizin-rich extract in therapy has been found to have many advantages in intensifying the treatment of metabolic disorders and diabetes [166].

The results of different preclinical studies indicate that phlorizin can effectively improve insulin resistance and lower serum glucose levels [167,168]. Additionally, Japanese researchers conducted an in vivo study to assess the delayed absorption of glucose in the intestines. They found that treating mice with oral phlorizin effectively reduced the postprandial rise in blood glucose levels [140]. These observations led to the synthesis of a significant phlorizin derivative called 3-(benzo[b]furan-5-yl)-2′,6′-dihydroxy-4′-methyl-propiophenone-2′-O-(6-O-methoxycarbonyl)-β-D-glucopyramide (T-1095) [142]. The experimental results demonstrated that the phlorizin derivative T-1095 significantly improved hyperglycemia in diabetic rats by inhibiting glucose reabsorption in the kidneys and reducing the expression of SGLTs and impaired renal glucose transporter 2 (GLUT2) expression [141].

Additionally, the administration of T1095 resulted in steady hyperglycemia and insulin resistance levels in the skeletal muscle of rats with streptozotocin-induced diabetes, which is remarkable [142].

Apple extracts and their juice have been found to contain between 11% and 36% total phlorizin, which can effectively lower levels of oxidized LDL [143].

Additionally, phloretin, the aglycone of phlorizin, triggered release in isolated coronary artery rings, which was not directly associated with endothelial function [169].

The effect of orally administered apple polyphenolic extracts on endothelial function was observed in 60 patients with borderline or mild hypertension through a randomized, double-blind, single-center, crossover study. Patients were administered a polyphenolic apple extract or placebo for 4 weeks. Consequently, significant acute enhancements in flow-mediated dilation of the brachial artery, which serves as a parameter for endothelial function, were demonstrated in different patients with hypertension. Contextually, NO status and endothelial function increased with the consumption of apples containing high concentrations of flavonoids [170].

A clinical trial involved 30 men with impaired fasting glucose who drank 500 mL of either treated (with invertase and glucose oxidase/CAT) or untreated apple juice. The sugar content, postprandial glycemic response, and venous serum insulin response all saw reductions of 21%, 68%, and 47% as a result of the enzymatic treatment. Without any adverse gastrointestinal side effects, the glycemic load was reduced by 74.6% [144]. Furthermore, a clinical study involved six high-risk women who consumed a low-sugar, high-fiber powder derived from immature apples to investigate its antihyperglycemic properties during a 50 g OGTT. The consumption of apple extract led to better glucose metabolism, characterized by lower postprandial glycemic response and higher urinary glucose excretion [170].

Due to the promising results of using SGLT2 inhibitors to treat diabetic cardiomyopathy, apple extract rich in phlorizin may be a beneficial supplement for addressing cardiac alterations associated with diabetes [171].

In addition, the inflammation that occurs after a heart attack (MI) plays a key role in restructuring the heart and its electrical pathways, impacting its pumping ability. The anti-inflammatory role of phloretin is attributed to its inhibition of the NLRP3/caspase-1/IL-1β pathway. Phloretin’s potential role in a rat model of MI was explored in a recent in vivo study, revealing its ability to inhibit the NLRP3/caspase-1/IL-1β pathway and upregulate Cx43 while limiting p38 phosphorylation, resulting in reduced susceptibility to ventricular arrhythmias. Furthermore, by inhibiting inflammation, phloretin reduced fibrosis and prevented HF. The inhibitory effects of phloretin on the NLRP3/caspase-1/IL-1β pathway were strongly supported by in vitro experiments on H9c2 cells. These findings indicate that phloretin may inhibit the NLRP3/caspase-1/IL-1β pathway, leading to the reversal of post-MI structural and electrical remodeling and thereby reducing the risk of HF [145]. Certain limitations affect the generalizability of these results. As an example, the inhibitoryphloretin effects on the activation of the NLRP3 pathway was observed after phloretin treatment [145]. An innovative study compared the cardioprotective effects of gala and Fuji apple juice in rats. The findings showed that both types of apple juice significantly prevented and protected against isoprenaline-induced cardiotoxicity by restoring myocardial biomarkers and hematological profile to control levels and inhibiting lipid peroxidation, maintaining antioxidant enzyme activities, and regulating cytokine levels [172]. Large-scale and long-term studies with numerous animals are crucial for obtaining valuable data to support clinical trials of these natural products. Additionally, exploring molecular mechanisms using various animal models and assessing multiple biochemical markers could enhance our understanding of the pathways implicated, potentially leading to improved myocardial function and reduced heart damage following myocardial ischemia. The outcomes shed new light on the advancement of specific preventive therapies for cardiovascular disorders.

### 4.2. Vitis vinifera L. Seed Extract

*Vitis vinifera* L. seed extract or grape seed extract (GSE) is rich in compounds that have been shown to have beneficial effects on many diseases, including cardiovascular disease. Stilbenoid resveratrol, found in grape skin, seeds, and wine, is a highly efficient bioactive compound known for its antioxidant and phytoestrogenic properties [173]. Resveratrol has been found to scavenge different oxidants, like hydroxyl radicals, superoxide, and hydrogen peroxide, and protects against cardiovascular damage [174]. An in vivo study examined how resveratrol contributes to the amelioration of HFpEF-induced administeredto mice. For a duration of four weeks, the mice were given either resveratrol (10 mg/kg/day, ig) or saline. Left ventricular hypertrophy, preserved ejection fraction, diastolic dysfunction, and pulmonary congestion were observed in HFpEF mice. In addition, neutrophils and macrophages were found to infiltrate the hearts of HFpEF mice to a greater extent. Additionally, HFpEF hearts exhibited heightened intracellular ROS levels. The expression of collagen-I, collagen-III, and transforming growth factor beta (TGF-β) mRNA was increased in HFpEF mice, while the protein expression of p-eNOS was decreased. By activating Sirt1, resveratrol can decrease Smad3 acetylation and transcriptional activity, potentially protecting against adverse cardiac remodeling in HFpEF according to the evidence [146].

Moreover, in an in vivo experiment involving rats with permanent ligation of the left coronary artery, resveratrol showed promise in providing a beneficial effect in HF. LV ejection fraction in the control group decreased significantly based on serial echocardiography, while LV end-systolic and end-diastolic volumes increased, and the myocardial infarct expanded compared to pretreatment values. There were considerable enhancements in those parameters observed in the resveratrol treatment group compared to the control group. Analysis of the LV pressure–volume loop demonstrated a significant enhancement in LV systolic function and AV coupling in R when compared to the control (*p* < 0.05). The measure of arterial stiffness, aortic pulse wave velocity, showed a significant decrease in R (389 ± 15 cm/s; *p* < 0.05). The study demonstrated that adding resveratrol to the diet over a long period of time improves cardiovascular health in congestive heart failure [147].

Based on this preclinical evidence, a clinical trial assessed the beneficial effects of resveratrol in patients with systolic heart failure (HFrEF) who were receiving standard therapy. Resveratrol treatment improved various parameters related to heart function, exercise tolerance, and clinical condition, while also reducing levels of inflammatory cytokines (IL-1 and IL-6). Echocardiography, a six-minute walking test, spirometry, quality-of-life questionnaire, lab test, and RNA profile analysis were conducted both before and after the study. Significant improvement was observed in the resveratrol-treated group in terms of systolic and diastolic left ventricular function, as well as global longitudinal strain, with an amelioration of exercise capacity, ventilation parameters, and quality of life. The treated group showed a decrease in cardiac biomarker levels, specifically NT-proBNP and galectin 3. The addition of resveratrol resulted in a significant reduction in inflammatory cytokines [175].

GSE also contains proanthocyanidins (PCs) that exhibit therapeutic, pharmacological, biological, and chemoprotective effects against free radicals and oxidative stress.

Interestingly, grape seed-derived PCs have demonstrated cardioprotective effects by affecting lysosomal and mitochondrial function [148]. In fact, when isoproterenol-treated rats were co-treated with PC, it reduced lysosomal enzyme activities in their cardiac tissue while increasing respiratory chain enzymes and mitochondrial activity [149]. The presence of PCs in GSE has been linked to antioxidant effects, with a dose-dependent relationship [176,177]. In mice, GSE showed significant protection against TPA-induced hepatic peroxidation, DNA fragmentation, cerebral lipid peroxidation, and peritoneal macrophage activation [176,177]. In this systematic review, the role of GSE was analyzed in several cardiovascular diseases. Nineteen studies were considered, and the weighted mean difference (WMD) and 95% confidence interval (CI) were calculated using a random-effect model. GSE supplementation significantly reduced heart rate (HR) (WMD: −2.20 mmHg, 95% CI: −3.79 to −0.60, I2 = 88.8%) (WMD: −1.25 bpm, 95% CI: −2.32 to −0. 19, I2 = 59.5%) and diastolic blood pressure (DBP) (WMD: −2.20 mmHg, 95% CI: −3.79 to −0.60, I2 = 88.8%), but had no significant effect on systolic blood pressure (SBP) (WMD: −3.55 mmHg, 95% CI: −7.59 to 0.49, I2 = 97.4%) or flow mediated dilatation (FMD) (WMD: 1.02%, 95% CI: −0.62 to 2.66, I2 = 92.0%). Subgroup analysis showed that the dose and duration of GSE supplementation and the nature of the study participants could provide sources of the heterogeneity between the studies. Significant non-linear relationships were found between diastolic blood pressure (DBP) and duration of GSE supplementation (*p* = 0.044) and its dose (*p* = 0.007). From this, it can be assumed that GSE may be beneficial for patients with or at risk of cardiovascular disease as it may have hypotensive and HR-lowering properties [178]. To assess the cardioprotective effect of PCs, a group of rats were given 100 mg of GSE orally in addition to their regular diet for 3 weeks. After this period, the rats were euthanized and perfused through Langerdoff. The analysis of GSE revealed a significant decrease in reactive oxygen species in the heart and an improvement in contractile regeneration after ischemia, suggesting cardioprotective action. The study findings suggest that GSE functions as an antioxidant in living organisms and its ability to block antideath signaling through inhibition of the transcription factor and pro-apoptotic gene JNK-1 and c-Jun contributes to its cardioprotective benefits [150]. In this paper, treatment with GSE in mice significantly reduced MI. In vitro, it inhibited apoptosis of H9C2 cells after culture in hypoxia, resulting in reduced expression of bcl-2 associated x protein (Bax) and increased expression of B cell lymphoma 2 (Bcl-2). Elevated expression of p-PI3K and p-AKT was revealed in the MI model in vivo and in vitro. Cardioprotection of GSE was controlled by LY294002, an inhibitor specific to the PI3K/AKT pathway. GSE has been found to enhance MI-induced cardiac dysfunction and remodeling and to inhibit cardiomyocyte apoptosis in hypoxic conditions by activating the PI3K/AKT signaling pathway [150].

### 4.3. Citrus bergamia Polyphenolic Fraction

The polyphenolic fraction of bergamot (BPF) has been shown to have a cardioprotective role. These polyphenols have an antioxidant role both in vitro and in vivo, as they reduce cholesterol, triglycerides, and glucose in serum, thus reducing anti-inflammatory action and consequently increasing endothelial function [179]. The inhibition of 3-hydroxy-3-methylglutaryl coenzyme A (HMGCoA) reductase is achieved by the compounds neoeriocitrin, neohesperidin, naringin, bruteridin, and melitidin, which are the important constituents of BPF [180,181,182].

#### 4.3.1. The Cardioprotective Role of BPF in Counteracting Doxorubicin-Induced Cardiotoxicity

A study investigating the protective role of BPF in doxorubicin-induced cardiac damage in rats revealed some interesting findings. BPF was found to strongly prevent ROS generation, apoptotic death of myocyte cells, and the overexpression of pro-autophagic mediators. Additionally, BPF was observed to counteract the decrease in resident cardiac stem cells (eCSCs) and stimulate the development of new myocytes in doxorubicin (DOXO)-treated rats [183]. The impact of BPF on the mitochondrial bioenergetics of pulmonary artery endothelial cells (PAECs) was examined in this study, focusing on its interaction with doxorubicin-induced cardiotoxicity. The cell viability of pAECs decreased by 50% after 24 h of treatment with different concentrations of DOX, but the negative effect of DOX was reversed when BPF was administered at increasing doses (100 µg/mL and 200 µg/mL). Inhibition of mitochondrial activity was observed at 0 µM DOX and remained unaffected by 200 µg/mL BPF. Conversely, the decrease in basal respiration and ATP production caused by 0.5 or 1.0 µM DOX was enhanced in the presence of 100 or 200 µg/mL BPF, respectively. After 24 h of cell recovery, the impaired mitochondrial parameters of oxidative metabolism caused by DOX were reactivated when treated with either 100 µg/mL or 200 µg/mL BPF on pAEC with 0.5 µM or 1.0 µM DOX, respectively [151].

#### 4.3.2. BPF Supplementation Counteracts Endothelial Dysfunction and Lipid Profile

In an experimental model of hypertension induced by unilateral renal artery ligation in rats, it was shown that dietary supplementation rich in bergamot polyphenols can counteract the hypertension-induced renal–cardiac syndrome, as BPF treatment prevents blood pressure elevation in renal artery ligation and treatment with deoxycorticosterone acetate (RAL DOCA-Salt) rats (*p* < 0.05) and has a protective effect on the volume of the contralateral kidney (*p* < 0.01). Additionally, BPF enhances cardiac tissue strain dysfunction through elevated Pk in displacement movement (*p* < 0.01) and decreased increased time to peak (T2P) in strain movement (*p* < 0.05) [152]. A systematic review gathered all studies on the impact of BPF on human lipid profile. Out of the ten studies, nine showed a notable decrease in total cholesterol, triglycerides, and LDL cholesterol, while one study demonstrated a significant decrease in only LDL cholesterol. Two studies concluded that there were no notable differences in any variable. Eight studies reported an increase in high-density lipoprotein (HDL) after intervention with bergamot in any form. These studies suggest a relationship between statin administration and both dose-dependent and potential synergistic effects. The potential synergistic effect may be due to bergamot’s ability to act on various levels, inhibiting HMG-CoA reductase, acyl-CoA cholesterol acyltransferase, and pancreatic cholesterol ester hydrolase. This would result in decreased cholesterol synthesis and enhanced fecal excretion of cholesterol. The promising results imply that bergamot supplements may offer an alternative therapeutic choice for dyslipidemia management, particularly for patients with moderate hypercholesterolemia, moderate cardiovascular risk, or those unable to tolerate traditional drug therapies [153].

### 4.4. Olea europaea L. Extract

Several studies have shown the positive impact of *Olea europaea* L. leaf extract (OLEX) on human health, supporting the benefits of the Mediterranean diet [184,185]. Virgin olive oil is a phytocomplex with around 100 non-glyceride substances that possess beneficial properties for human health, such as antioxidants, anti-inflammatory agents, and antimicrobials [186]. The European Food Safety Authority (EFSA) has classified extra-virgin olive oil as a functional food. Scientific research suggests that the polyphenols found in olive oil help safeguard blood lipids against oxidative stress [187].

The correlation between aging, olive oil usage, and bioclinical variables was supported by trials in the Athens metropolitan area (Attica) and Mediterranean islands. To promote healthy aging, a key recommendation is to exclusively use olive oil for cooking [186].

The bioactive compounds in OLEX, especially the lipophilic and hydrophilic phenols, have significant antioxidant and nutraceutical effects. These compounds are synthesized by the plant to protect against attacks by pathogens and insect injuries [176].

**Figure 3:**
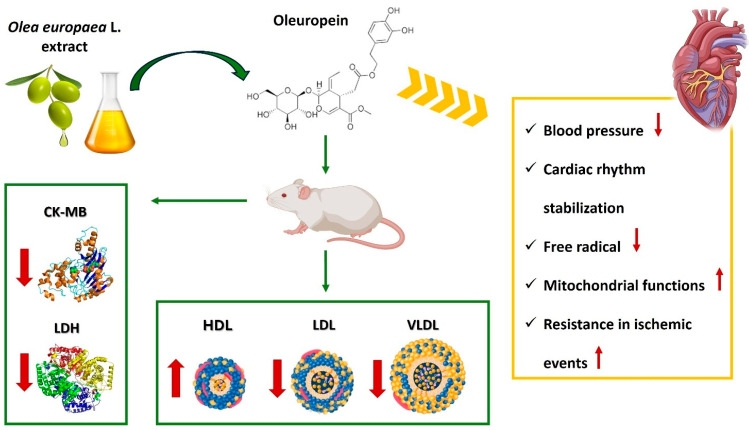
Oleuropein-mediated heart benefits obtained from olive leaves and fruits after administration to rats. OL promotes the reduction of creatinine kinase MB (CK-MB) isoenzyme levels and lactate dehydrogenase (LDH) levels, effectively decrease LDL, VLDL cholesterol levels, while increasing HDL cholesterol in vivo. These effects promote the reduction of blood pressure by vasodilation, stabilization of heart rhythm, protection of cardiomyocytes from oxidative stress by lowering the damage caused by free radicals, improvement in mitochondrial function and resistance of cardiomyocytes to ischemic events. Creatine kinase myocardial band (CK-MB); lactate dehydrogenase (LDH); high-density lipoprotein (HDL); low-density lipoprotein (LDL); very-low-density lipoprotein (VLDL).

The beneficial properties of the secoiridoid oleuropein (OL) and its derivatives, such as hydroxytyrosol (HT), tyrosol, oleacein, and oleocanthal, have been recently discovered in the field of nutraceuticals [187,188]. The high HT and OL bioavailability plays a crucial role in in vivo antioxidant activity: both compounds have been found to rely on continuous absorption in order to sustain their metabolic and pharmacokinetic effects (Figure 3) [189].

Secoiridoids act as antioxidants, scavenging free radicals and chelating metals to disrupt radical chains and inhibit the formation of phenolic peroxyl radicals, resulting in increased structural stability [190,191]. The extraction process of olive drupes specifically releases these compounds. The higher concentration of HT in olive oil and drupes is a result of chemical and enzymatic reactions during fruit maturation [192]. OL aglycone has been proven clinically to enhance disease condition and platelet aggregation by interacting with tau protein, amyloid β-protein (Aβ1–40), and alpha-synuclein [193].

Furthermore, by activating the mitogen-activated protein kinase/extracellular signal-regulated kinase signaling pathway (ERK/MAPK), OL (1 μM) or the secoiridoid ligstroside (10 μM) enhances insulin secretion in pancreatic β-cells, both in response to glucose stimulation and under basal glucose uptake regulation [194]. Secoiridoids, extracted through purification processes, play a crucial role in preventing the development of atherosclerosis in later stages. At a concentration of 100 μM, OL has been observed to hinder the growth of vascular smooth muscle cells [160].

The atherosclerotic process and plaque instability in cardiovascular disease can be improved by oleacein (10–20 μM), which inhibits various molecular targets, including high mobility group protein 1, matrix metalloproteinase 9, matrix metalloproteinase 9–neutrophil gelatinase-associated lipocalin complex, and tissue factor [154].

The inhibitory effects of luteolin, a widely found flavone in olive leaves, on ROS and TNF-α have been demonstrated in several studies, suggesting its potential for anti-obesity benefits, improved vascular function, and reduced body fat weight [195].

The positive impact of polyphenols on dyslipidemia in non-alcoholic fatty liver disease (NAFLD) was confirmed through a study on Olea polyphenols [157]. Evidence suggests that lutein may have cardioprotective properties against conditions such as coronary artery disease, atherosclerosis, and heart failure [155].

Luteolin-7-O-glucoside, a glycosidic form of luteolin found in Olea europaea leaves, can regulate hepatic lipid metabolism, resolve inflammation, and regulate metabolic diseases related to diet [157].

Oleocanthal, primarily found in olive oil fruit, has demonstrated anti-inflammatory properties by inhibiting Cox-1 and Cox-2 in a dose-dependent manner, making it a natural non-steroidal anti-inflammatory (FANS) [196].

Along with this relevant activity, it displays the most potent cytotoxic and anti-proliferative properties against various cancer cell lines when compared to other secoiridoids present in olive oil [197]. Oleacein, a byproduct of OL sourced from olive tree leaves, displays antioxidant, anti-bacterial, and anti-inflammatory actions. Specifically, through antiviral testing, it was discovered that it effectively regulates protein expression in the SARS-CoV-2 virus, enhancing the infection’s prognosis [198]. The role of olive leaf extract in fighting various viruses was examined in a triple-blinded clinical trial on hospitalized COVID-19 patients. A new therapeutic treatment approach has gained attention recently, involving capsules containing 30 percent OL to control COVID-19, as mentioned earlier. By reducing the erythrocyte sedimentation rate and C-reactive protein levels, this biophenol increases body temperature, respiratory rate, oxygen saturation, and blood pulse rate, resulting in shorter hospital stays. Some restrictions applied to the study: patients admitted to the intensive care unit or who passed away were excluded. It is recommended that mortality and the necessity for intensive care be regarded as study results. Individuals without preexisting conditions were exclusively part of this study, whereas numerous patients had a background of chronic illness. The findings were not applicable to the entire population. Additionally, this study had limitations, including not examining other inflammatory markers like IL-6 and D-dimer on the final day. To determine the efficacy of olive leaf extract in treating COVID-19 patients, further studies with larger samples are needed. It is important to factor in the impact of confounding variables such as underlying diseases [156].

The concentration of biophenols in Olea leaf extract (OLEX) is affected by various agronomic factors, including the origin of the olives, irrigation method, cultivar, ripeness, and extraction conditions [199].

The presence of different olive cultivars in the Calabria region can lead to variations in the quantity and quality of bioactive compounds extracted from the leaves, potentially influencing the properties of olive products and the formation of active metabolites. Optimal extraction method selection and definition are crucial for bioactive compounds in industrial processes to maximize yield and minimize costs. Traditional extraction methods using organic solvents have been used to extract olive leaves [157].

Due to their high production and disposal costs, research has increasingly focused on valorizing and reusing EVOO by-products as an environmental solution.

The by-products of olive mill wastewaters and olive oil leaves contain bioactive compounds with beneficial properties, making them a potential ingredient for functional foods and nutraceuticals [200,201]. The antioxidant activity of the extract concentrated in phenolic compounds (OLECp) was verified for scavenging hydroxyl radicals and DPPH using electron paramagnetic resonance (EPR). The anti-inflammatory activity and potential benefits of oleic acid (OA) in reducing lipid accumulation were investigated in an in vitro model of non-alcoholic fatty liver disease (NAFLD) using McA-RH7777 cells. Based on both scavenging free radicals (IC50: 2.30 ± 0.18 mg/mL) and decreasing signal area in the EPR spectra, OLECp proved to be highly effective in neutralizing hydroxyl radicals and DPPH [157]. Moreover, OL effectively prevented the onset of cardiomyopathy in rats exposed to DOXO [202]. DOXO is a commonly utilized chemotherapeutic agent and is classified as an anthracycline. However, one of the main restrictions associated with its clinical use is cardiotoxicity, which can lead to congestive heart failure. Indeed, anthracyclines have been found to increase the risk of heart failure in approximately 15–17% of patients, causing damage to the heart at various levels [203].

Other natural derivatives like BPF and *Cynara cardunculus* exhibit similar beneficial traits to OL. An investigation was conducted on the effects of these three plant derivatives using an in vitro cardiotoxicity model. By treating rat embryonic cardiomyoblasts (H9c2) with DOXO, this model was developed to reduce cell death, oxidative damage, and calcium ion leakage from the endoplasmic reticulum and increase lipid content [204]. A study conducted by Granados et al. demonstrated the amelioration of mitochondrial dysfunction in breast cancer rats, prompting the use of HT drugs to alleviate chronic cardiac toxicity from DOXO [205].

Furthermore, HT treatment effectively decreases mitochondrial swelling and vacuolization, preserving the integrity of mitochondrial electron transport chain complex III (METC) following DOXO. The beneficial properties of olive oil were investigated for their effects on heart failure caused by post-myocardial infarction (PMI) induced by coronary artery constriction in rats. To conduct the study, the animals were divided into sham and bound groups. They were provided with either regular food, olive oil (10% *w*/*w*), or corn oil (10% *w*/*w*). The animals’ behavior was monitored over a 16-week period. The study revealed that the plasma levels of TNF-α were noticeably reduced in the LOO group after 4 weeks of PMI, in comparison to the LRC group.

The myocardial redox ratio (reduced glutathione/oxidized glutathione) decreased by 44.4%, 16.4%, and 36.9% at 4 weeks post-mortem interval in the LRC, LOO, and LCO groups, respectively, compared to the initial values. At 16 weeks post-intervention, the redox ratio in the CRL and LCO groups worsened even more. The LRC, LOO, and LCO groups showed significant increases in lipid hydroperoxide formation of 137.4%, 14.6%, and 97.1%, respectively, at 4 weeks PMI [158]. Age-related declines in protein expression, infarct size, and apoptosis in cardiomyocytes are significantly influenced by HT. Rats treated with tyrosol experienced decreased infarct size and improved myocardial function compared to untreated animals [206,207].

Additional research on myocardial ischemia–reperfusion in rats found that OL could lower CK-MB and LDH levels by affecting the ERK pathway and inhibiting p53, p-MEK, and p-ERK (Figure 3) [208]. The nephrotoxic effects of cisplatin can be attenuated in mice by OL, which inhibits p53 and ERK signaling to protect against kidney injury [209]. Moreover, these findings suggest that OL protection is linked to decreased levels of total cholesterol and triglycerides in the reperfused myocardium, factors that promote the onset of infarction. Purified secoiridoids may have a significant effect on hypertension, a key cardiovascular disease risk factor, as indicated by in vitro studies showing antihypertensive or vasodilator properties. Oleacein has been proven to inhibit the activity of angiotensin-converting enzyme (IC50 26 μM) and directly relaxes vascular smooth muscle cells [160]. Health effects were observed in the early and advanced stages of atherosclerosis in in vitro studies using purified secoiridoids [160].

The formation of foam cells is improved by oleacein at a concentration of 50 μM, as it decreases the expression of receptors (scavenger A1 receptor, CD36, lectin-like oxLDL receptor) responsible for binding and absorbing oxidized LDL into macrophages [159]. On the other hand, OL at concentrations between 50 and 100 μM inhibited the expression of endothelial leukocytes, thus reducing the adhesion of monocytic cells to vascular endothelial cells. In advanced stages of atherosclerosis, OL (100 μM) has been reported to inhibit the proliferation of vascular smooth muscle cells, as well as platelet aggregation [160].

Furthermore, *Olea europaea* L. leaves yielded aqueous and ethanolic extracts with remarkable antioxidant potential in vitro, and their use in hypercholesterolemic mice demonstrated their effectiveness in reducing cholesterol levels. The levels of serum total cholesterol, triglycerides, and lipoproteins (HDL, LDL and very-low-density lipoprotein (VLDL)) were also evaluated. The findings showed that mice treated with both extracts had lower levels of total cholesterol (246.6 and 163.4 mg/dL) and LDL (150.16 and 81.28 mg/dL) compared to the hypercholesterolemic group. In addition, mice treated with the aqueous extract (200 mg/kg body weight) experienced a significant decrease in triglyceride and VLDL levels (Figure 3) [210].

Furthermore, the prevention of endoplasmic reticulum (ER) stress provides evidence of the cardioprotective effects of OLEX and HT. The research suggests that HT administration can prevent apoptosis triggered by ER stress in H9c2 cells exposed to hypoxia by suppressing the expression of GRP78 and CHOP at mRNA and protein levels [211].

The protective effect of OL on human vascular progenitor cells (hVPCs), which are prone to depletion from ROS-mediated oxidative stress, has been recognized recently. OL has been shown to directly prevent oxidative stress in conditions like hypertension by scavenging O2, highlighting its effectiveness [212].

A study was conducted to compare the health benefits of oleocanthal- and oleacein-rich extra-virgin olive oil (EVOO) with ordinary olive oil (OO) in individuals aged 40–65 with prediabetes and obesity. The focus of this study was to substitute regular oil with EVOO or OO, with no changes to physical activity or diet. Inflammation was the primary result identified, followed by subsequent changes in body weight, oxidative status, lipid profile, and glucose management. The study included 91 patients (33 men and 58 women) and used an ANCOVA model for statistical analysis. The analysis was adjusted for parameters such as gender, age, and treatment administration sequence. After EVOO treatment, there was a significant increase in total antioxidant status, a decrease in lipid and organic peroxides compared to OO treatment (*p* < 0.05), and a reduction in interferon-γ with inter-treatment differences (*p* < 0.041). Weight, BMI, and blood glucose decreased significantly (*p* < 0.05) after EVOO treatment, indicating its effectiveness. In conclusion, it was determined that EVOO treatment, rich in polyphenols like oleocanthal and oleacein, impacts inflammatory and oxidative responses in varying ways for individuals with obesity and prediabetes [161].

In the PREDIMED trial, 7216 men and women (aged 55–80 years) with high cardiovascular risk were included. The study analyzed the random assignment of trial participants to three diets: Mediterranean diet with nuts or EVOO, and a low-fat control diet. The average intake of total olive oil was 56.9 g/day in participants allocated to the highest tertile, compared with 21.4 g/day in participants assigned to the lowest tertile. A decrease of 10% in the risk of cardiovascular events increasing for each 10 g/day in EVOO intake was observed. The recommended daily intake of olive oil is 25–50 mL, with a maximum of 0.9 mg of oleocanthal (equivalent to 5–10 teaspoons). The concentrations of oleocanthal in a regular daily portion of EVOO (25–50 mL) are believed to have restricted effects on inflammation and oxidation in vivo based on this assumption [184].

Evidence suggests that incorporating olive oil into one’s diet can lead to a decrease in mortality rates, particularly from cardiovascular diseases. A thorough analysis has been conducted on the data from the Molisani study, examining mortality in the cohort from March 2005 to 31 December 2020. The aim of the study was to analyze the connection between olive oil consumption and mortality in 22,892 men and women in Italy over a 13.1-year follow-up period.

The quality of the Mediterranean diet was evaluated using a score of 188 items collected between 2005 and 2010, with olive oil consumption standardized to 10 g per tablespoon (tbsp). By employing Cox proportional risk models adjusted for various factors, such as diet quality, and hazard ratios (HRs) and confidence intervals (CIs) were estimated at 95%. Through the analysis of estimate variability and associated *p*-values, we explored the potential involvement of metabolic, inflammatory, renal, and cardiovascular biomarkers in the relationship between olive oil consumption and mortality. Indeed, the different HR values were in the range of 0.80 (0.69–0.94), 0.77 (0.59–0.99), 0.75 (0.58–0.97), and 0.97 (0.73–1.29) for all-cause mortality, cardiovascular causes, cancer, and other causes associated with a high consumption of olive oil of more than 3 tbsp/day.

Olive oil consumption is linked to decreased rates of cardiovascular mortality, irrespective of diet quality, with diseases and cancer mortality reduced by 21.2% and 13.7%, respectively [213]. The leading causes of CVD (cardiovascular disease) and other disorders are often obesity and overweight. OLEX-containing phenols have been shown to positively impact the development of hypertension and dyslipidemia. The purpose of one study was to assess the alterations in blood lipid profiles in overweight/obese subjects with slightly elevated cholesterol levels following an 8-week treatment with OLEX. The study included 77 overweight/obese adults who were randomly assigned to receive either OLEX or placebo for 8 weeks. At both the 4-week and 8-week marks of diet therapy, blood cholesterol, blood pressure, glucose, oxLDL, and insulin levels were examined. After 8 weeks, the liver function parameters were monitored. Blood lipid levels did not show significant differences after 4 or 8 weeks of OLEX diet supplementation compared to placebo (*p* < 0.05). The intervention groups did not differ significantly in terms of oxLDL, glucose, blood pressure, insulin levels, or liver function parameters (*p* < 0.05). To conclude, the incorporation of OLEX for 8 weeks did not result in significant modifications to blood lipid profiles [162]. It is necessary to address certain drawbacks of the study in question. At first, there were minor variances in baseline values for certain outcome measures between the two intervention groups. Nevertheless, these variances did not affect the conclusions due to statistical analyses adjusting for initial values. Furthermore, the alterations in blood lipids, blood pressure, and glucose levels were minimal and seemed to lack clinical significance. Third, we were unable to determine the intake of trans-fatty acids from the food records in the study. As there were no substantial modifications in the consumption of primary food elements, we do not believe that the intake of trans-fatty acids influenced our findings. Fourth, the dosage in the current study was determined by a previous study involving around 100 mg of oleuropein in OLE, which demonstrated efficacy after 12 months of supplementation [162].

## 5. Conclusions

In summary, this extensive review evaluated the impact of lifestyle changes, nutrition, and nutraceutical supplementation on preventing and supporting heart failure treatment, highlighting potential candidates for future clinical studies in HF patients.

Despite the lack of evidence on lifestyle variation in individuals with HF, current research indicates the need to examine different aspects in the daily management of HF. The DASH diet offers clear benefits in terms of nutrition, but the effectiveness of the Mediterranean diet is still inconclusive. In terms of salt consumption limitation, it is more advisable to moderately reduce rather than severely restrict. Physical activity and cardiac therapy are helpful for HF, but the exact type and duration are not clearly defined. While fluid restriction is often recommended, several brief clinical studies have indicated no alleviation of congestion.

Furthermore, changes in the oxidative environment and inflammation significantly impact the clinical development and progression of HF.

The presence of multiple comorbidities leads to systemic inflammation, causing HFpEF. Inflammation causes a decrease in nitric oxide (NO) availability, ROS production, and activation of cyclic guanosine monophosphate-dependent serine/threonine protein kinase (cGMP-dependent PKG). Consequently, there is a development of myocardial hypertrophy and diastolic dysfunction. Moreover, patients with HFrEF show reduced NO bioavailability and cGMP levels, leading to impaired vasodilation, decreased aerobic capacity, and muscle strength.

Endothelial dysfunction modulation is being reevaluated due to new insights into how failing myocardial cells can be stimulated through endogenous biomolecular mechanisms, particularly through the impact of nutritional sources with active ingredients.

Preclinical and clinical studies have demonstrated that natural bioactive compounds isolated from *Olea europaea* L., *Malus domestica*, *Vitis vinifera* L., and *Citrus bergamia* (Risso and Poiteau) can effectively decrease oxidative stress, inflammation, endothelial dysfunction, hyperlipidemia, and hyperglycemia. As a result, these derivatives offer promising and innovative approaches to treating HF. To sum up, an increasing amount of clinical data show that consuming the right dosages of specific nutraceuticals may result in better self-perceived quality of life and improvements in functional parameters such as EF, stroke volume, and cardiac output among heart failure patients, with few to no side effects. However, further assessment and data from the Cardiovascular Outcome Trial are necessary to validate the effectiveness of nutrition and nutraceuticals in HF prevention and treatment. More comprehensive studies can produce more trustworthy findings by reducing error margins and deviation levels, thereby minimizing the likelihood of inaccurate results.

## Figures and Tables

**Table 2:** List of the main effects exerted by lifestyle modifications in HF management, starting from exercise and nutrition to addiction and HF.

Exercise Training and HF Management		
Clinical Trials	Properties	Ref.
ESC guidelines recommend physical activity for all qualified patients	Improving the level of exercise, decreasing hospitalization and quality of life	[32]
AHA/ACC HF 2022 guidelines for exercise program	Improvement in physical evidence, functional status, and quality of life	[33,34]
Impact of ET related to VO2	Improves exercise effectiveness with a value corresponding to 15–17% Increase in time spent on exercise, work weight, quality of life, and distance traveled in 6 min	[35]
Meta-analysis on the effectiveness of ET, rehabilitation on heart function, and pulmonary circulation measurements in HF subjects with HFpEF	The mitral E/e relationship is significantly reduced in the post-training period, evidenced by five clinical studies	[36]
The comparative effectiveness of IT and CT on the cardiac and respiratory fitness and exercise compliance of subjects with HF	Increase the highest oxygen absorption, 6 MWD, and left ventricular ejection fraction No significant changes in respiratory exchange relationship, resting heart rate, or slope of respiration corresponding to CO_2_	[37]
Relationship between the efficacy of aerobic exercise on maximal VO2	Aerobic exercise gradually increased the VO2 peak Increased health-related quality of life in subjects with HFpEF	[38]
Baduanjin exercise	Positive changes in QoL, exercise ability, and physical decline	[39]
**Exercise-Based Cardiac Rehabilitation**		
**Clinical Trials**	**Properties**	**Ref.**
Exercise associated with cardiac rehabilitation (LV value that is <40%) according to CROSH-FH clinical study	Negative results on mortality and hospitalization frequency of patients Increased quality of life and ability to perform exercise	[40]
HF-ACTION clinical study	Cardiac rehabilitation adherence by exercise was <30% in the long term	[35]
**HF Nutrition**		
**Clinical Trials**	**Properties**	**Ref.**
Lethality in underfed patients	Increase to 68.9%	[41]
Vitamin D addiction	No improvement	[42,43]
DASH and Mediterranean diet	Lower risk of predisposition to HF Decline of rate of hospitalizations	[33,44,45]
Antioxidant and potassium concentration in DASH diet	Reduction in HF hospitalizations	[33,44,45]
Low-sodium DASH diet	Decrease in hardness, blood pressure Improvements in ventricular diastolic function Improvement in oxidative stress	[44,45]
**Prevention of Excessive Salt and Fluid Consumption**		
**Clinical Trials**	**Properties**	**Ref.**
Dietary sodium restriction (800 mg/day to 3000 mg/day) SODIUM-HF: Restricting dietary salt consumption (up to 1500 mg per day in subjects with HF)	Frequency of hospitalization or mortality in patients with HF remains unmodified No improvement in hospitalizations or emergency room admissions with standard therapy for one year	[46]
Low-fluid diet for patients with low blood sodium levels in SALT-HF clinical study (<135 mmol/L)	Improved quality of life	[47]
Correlation between chronic dehydration (hypohydration) and HF in ARIC study	39% of increased risk of developing HF corresponding to a 1% reduction in body water weight	[48]
**Body Weight Management**		
**Clinical Trials**	**Properties**	**Ref.**
“Obesity paradox”	Positively influenced the prognosis in overweight and mildly obese individuals (BMI between 30 and 34.9 kg/m^2^)	[49]
Bariatric surgery	Greater weight reduction: a loss of 18.8 kg more after one year and 22.6 kg more after two years Decrease in the development of heart failure	[50]
Efficacy of ASV treatment implemented on cardiac function and remodeling	Negative result for adaptive servo-ventilation therapy on left ventricular ejection fraction Negative result for plasma BNP concentrations over a maximum 24-week period Improvements in patients’ quality of life and clinical status	[51]
CPAP in HFrEF, according to clinical study	Benefits on markers of apnea, hypopnea, frequency of awakenings during the night, systolic pressure during the day, and heart rate	[52]
**Link Between Addiction and HF Management**		
**Clinical Trials**	**Properties**	**Ref.**
High alcohol ingestion	Enhanced HF progression Induction of dilated cardiomyopathy	[53]
Patients with alcoholic cardiomyopathy	Reduced the risk of death from cardiovascular causes, coronary heart disease, and stroke development by 14–25% with consumption of 2.5–14.9 g/day of alcohol	[54]

HF: heart failure, ESC: European Society of Cardiology, ET: exercise training, VO2: maximal oxygen consumption, HFpEF: heart failure with preserved ejection fraction, QoL: quality of life, SALT-HF: Strict Allowance of Fluid Therapy in Hyponatremic Heart Failure, LV: left ventricular diastolic function, 6 MWD: 6 min walking distance IT: interval training, CT: continuous training, DASH: Dietary Approaches to Stop Hypertension, ARIC: Atherosclerosis Risk in Communities, BMI: body mass index, ASV: adaptive servo-ventilation, BNP: brain natriuretic peptide, CPAP: continuous positive airway pressure, HFrEF: heart failure with reduced ejection fraction.

**Table 3:** Nutritional supplementation with ferric carboxymaltose (FCM), polyunsaturated fatty acids (PUFAs), and CoQ10 in the prevention and therapeutic support of HF.

Nutritional Supplementation in Prevention and Therapy Support for HF		
Clinical Trials	Properties	Ref.
Relationship between plasma vitamin C levels and HF incidence	Positive association Lower risk of HF	[95]
PUFA supplementation	Positive outcomes for HF patients	[93]
Doses relevant to the clinical context of DHA	Mitigated the risk of mitochondrial permeability transitions triggered by Ca^2+^ Reduced stress	[93]
**Supplementation with Ferric Carboxymaltose (FCM)**		
**Clinical Trials**	**Properties**	**Ref.**
A single-arm study in 2006 examining IV iron treatment in 16 patients with HF and systolic HF for 12 days	Improvement in the 6 MWD test and MLHFQ	[98]
A randomized study of 40 HF patients with an EF of 40% receiving IV 200 mg sucrose per week for 5 weeks	Patients who were given IV iron exhibited lower levels of NT-pro-BNP Improved functional capacity Reduced number of hospitalizations over the course of 6 months	[99]
A 2008 randomized study of 35 patients with an EF of 45%, anemia, and evidence of iron deficiency carried out with a total of 1400 mg of iron sucrose	No significant differences in peak oxygen consumption or treadmill exercise duration (*p* = 0.08 for both) Significant improvements in NYHA functional class Improvement in global patient assessment	[100]
Patients received 200 mg IV of FCM in the FAIR-FH study, with a maintenance dose every 4 weeks	50% versus 28% of patients showed improvement with FCM	[101]
The 6 MWD and questionnaire	The difference in serum ferritin levels was 246 ng/mL The difference in Hb concentration was 0.5 g/dL Normal Hb concentration (12.5 g/dL at the end of the trial)	[102]
The CONFIRM-HF study enrolled 304 symptomatic outpatients with HF with left ventricular EF ≤ 45% and iron deficiency randomized 1:1 to treatment with FCM or saline and followed up for 52 weeks	Treatment with FCM significantly improved the 6 MWD test at week 24 Improvements in NYHA class and other functional/QoL measures with statistical significance A significant reduction in the risk of hospitalization for worsening HF (HR 0.39, CI 95% 0.19–0.82; *p* = 0.009)	[102,103]
**Supplementation with Polyunsaturated Fatty Acids** **(PUFAs)**		
**Clinical Trials**	**Properties**	**Ref.**
A meta-analysis involving nearly 80,000 heart failure patients	Ω-3 did not have a substantial impact on heart failure admissions or cardiovascular mortality A slight but significant effect on rehospitalizations	[104]
MESA was conducted to assess the role of Ω-3 in preventing heart failure, including 6562 participants (52 females) with a duration of 13 years	Higher plasma percentage of EPA, measured and expressed as %EPA, was related to a lower risk of HF, in particular HFrEF and HFpEF, which was also valid for the other Ω-3, in particular for the combination % EPA + % DHA	[105]
Clinical studies carried out in obese patients with HFpEF	Consuming a diet rich in UFAs (MUFAs and PUFAs) improved diastolic function and cardiorespiratory efficiency, leading to increased fat mass	[106]
The EPA/AA ratio was studied in 577 HF patients divided into 2 groups, and its correlation with HF patient mortality was discovered	No differences were found in parameters such as BNP levels, blood pressure, and LVEF Variations in cardiac mortality rates existed, and they were inversely related to the EPA/AA ratio The ratio could independently predict cardiac mortality in patients with HF	[107]
12 RCTs were incorporated into a study that included 81,364 patients and their follow-up periods of 6 months to 6.2 years	The randomized groups had no significant differences in the rate of first HF hospitalization The placebo groups showed no significant disparity in cardiovascular mortality, with 990 cases for Ω-3 and 948 cases for the control group Ω-3 patients experienced a significantly decreased rate of recurrent HF hospitalization (1432 placebo and 1330 omega-3; RR 0.91; 95% CI 0.85–0.98, *p* = 0.02; I2 = 71%)	[108]
The serum ALA level of patients with HF was studied, specifically revealing 85 cardiovascular deaths, 140 all-cause deaths. and 141 first HF hospitalizations	The risk of composite deaths and first HF decreased significantly for patients in the 3 upper quartiles (Q2–Q4) compared to those in the lowest quartile of ALA in serum (Q1) using Cox regression analyses (HR: 0.61; 95% CI: 0.46–0.81) The decrease in all-cause death was statistically significant (HR: 0.58; 95% CI: 0.41–0.82), cardiovascular death (HR: 0.51; 95% CI: 0.32–0.80), first HF hospitalization (HR: 0.58; 95% CI: 0.40–0.84), and the composite of cardiovascular death and HF hospitalization (HR: 0.58; 95% CI: 0.42–0.79) During the midterm follow-up, patients with the highest ALA levels had a better prognosis than those with lower levels	[109]
**Supplementation with Coenzyme Q10 (CoQ10)**		
**Clinical Trials**	**Properties**	**Ref.**
Study conducted using CoQ10 400 mg before PPCI and 200 mg twice daily for three days after PPCI vs. placebo	Plasma level of antioxidant biomarkers was similar in both groups at 6 and 24, h; at 72 h, there were significant ameliorations in the group treated with CoQ10	[110]
Trial of patients with HFrEF	Group treatment with CoQ10 decreased hospitalization over the control	[111]
Q-SYMBIO trial: 420 participants administered two years’ 100 mg of CoQ10 in addition to standard therapy	CoQ10 in addition to standard therapy resulted in a decrease of cardiovascular death, HF hospitalizations, mechanical support or cardiac transplant, death from cardiovascular causes, and all-cause mortality	[112]
Subgroup analysis of the Q-SYMBIO randomized double-blind trial	After two years, decreased mortality by 53% in the CoQ10 group compared to placebo Hospitalization due to worsening HF significantly decreased in the CoQ10 group (3%) compared to the placebo group (13%)	[113]
Pilot randomized, double-blind trial placebo-controlled trial in HFpEF patients over 55 old	Supplementation with CoQ10: no clinical benefit was observed, due the fact that HFpEF exhibits different clinical phenotypes	[114]

HF: heart failure, PUFAs: polyunsaturated fatty acids, DHA: docosahexaenoic acid, MLHFQ: Minnesota Living with Heart Failure, 6 MWD: 6 min walking distance, NT-pro-BNP: N-terminal pro-B-type natriuretic peptide, IV: intravenous injection, NYHA: New York Heart Association, CI: confidence interval, FCM: ferric carboxymaltose, QoL: quality of life, Hb: hemoglobin, Ω-3: omega-3, MESA: Multi-Ethnic Study of Atherosclerosis, HFrEF: heart failure with reduced ejection fraction, HFpEF: heart failure with preserved ejection fraction, EPA: eicosapentaenoic acid, MUFAs: monounsaturated fatty acids, BNP: brain natriuretic peptide, LVEF: left ventricular ejection fraction, RCTs: randomized control trials, ALA: alpha-linolenic acid, Cox: cyclooxygenase enzyme, CoQ10: coenzyme Q10, PPCI: primary percutaneous coronary intervention.

**Table 4:** Recent preclinical and clinical evidence in nutraceutical supplementation and HF.

Recent Preclinical and Clinical Evidence in Nutraceutical Supplementation and HF		
Clinical Trials	Properties	Ref.
Intake of specific nutraceuticals	Improvements in functional parameters like stroke, ejection fraction, volume, and cardiac output in HF patients, with minimal side effects reported in clinical studies through anti-ischemic, anti-inflammatory, antioxidant, and anti-platelet effects	[137,139]
**Malus domestica Derivatives**		
**Clinical Trials**	**Properties**	**Ref.**
Japanese researchers conducted an in vivo study to assess the delayed absorption of glucose in the intestines treating mice with oral phlorizin	Reduction in postprandial rise in blood glucose levels	[140]
Administration of the phlorizin derivative T-1095 in diabetic rats	Significantly improved hyperglycemia by inhibiting glucose reabsorption in the kidneys. Reducing the expression of SGLTs and impaired renal glucose transporter 2 (GLUT2) expression	[141]
Administration of T1095 in rats with streptozotocin-induced diabetes, which is remarkable	Resulted in steady hyperglycemia and insulin resistance levels in the skeletal muscle of rats	[142]
Apple extracts and their juice have been found to contain between 11% and 36% total phlorizin	Effectively reduction the levels of oxidized LDL	[143]
A clinical trial involved 30 men with impaired fasting glucose who drank 500 mL of either treated (with invertase and glucose oxidase/CAT) or untreated apple juice	The sugar content, postprandial glycemic response, and venous serum insulin response saw reductions of 21%, 68%, and 47%, respectively, as a result of the enzymatic treatment No adverse gastrointestinal side effects, the glycemic load was reduced by 74.6%	[144]
Phloretin’s potential role in a rat model of MI was explored in a recent in vivo study	Inhibited the NLRP3/caspase-1/IL-1β pathway Upregulated Cx43 while limiting p38 phosphorylation Reduced susceptibility to ventricular arrhythmias (VAs) Phloretin reduced fibrosis and prevented HF by inhibiting inflammation	[145]
The inhibitory effects of phloretin on the NLRP3/caspase-1/IL-1β pathway were strongly supported by in vitro experiments on H9c2 cells	Phloretin may inhibit the NLRP3/caspase-1/IL-1β pathway, leading to the reversal of post-MI structural and electrical remodeling, thereby reducing the risk of VAs and HF	[145]
***Vitis vinifera* L. Seed Extract**		
**Clinical Trials**	**Properties**	**Ref.**
In vivo study examining resveratrol	Amelioration of HFpEF-induced cardiac remodeling Decreases Smad3 acetylation and transcriptional activity Potential protection against adverse cardiac remodeling in HFpEF	[146]
In vivo experiment involving rats with permanent ligation of the left coronary artery	Adding resveratrol to the diet over a long period of time improved cardiovascular health in congestive heart failure	[147]
Grape seed-derived PCs	Demonstrated cardioprotective effects by affecting lysosomal and mitochondrial function. Isoproterenol-treated rats were co-treated with PC: reduced lysosomal enzyme activities in their cardiac tissue while increasing respiratory chain enzymes and mitochondrial activity	[148,149]
To assess the cardioprotective effect of PCs, a group of rats were given 100 mg of GSE orally in addition to their regular diet for 3 weeks	Significant decrease in reactive oxygen species in the heart Improvement in contractile regeneration after ischemia, suggesting a cardioprotective action GSE functions as an antioxidant in living organisms and its ability to block antideath signaling through inhibition of the transcription factor and pro-apoptotic gene JNK-1 and c-Jun contributes to its cardioprotective benefits	[150]
***Citrus bergamia* Polyphenolic Fraction**		
**Clinical Trials**	**Properties**	**Ref.**
The impact of BPF on the mitochondrial bioenergetics of pulmonary artery endothelial cells (PAECs) was examined, focusing on its interaction with doxorubicin-induced cardiotoxicity	The cell viability of PAECs decreased by 50% after 24 h of treatment with different concentrations of DOX The negative effect of DOX was reversed when BPF was administered at increasing doses (100 µg/mL and 200 µg/mL)	[151]
Dietary supplementation rich in bergamot polyphenols in experimental model of hypertension induced by unilateral renal artery ligation in rats	Counteracted the hypertension-induced renal–cardiac syndrome, as BPF treatment prevents blood pressure elevation in renal artery ligation and treatment with deoxycorticosterone acetate (RAL DOCA–salt) rats (*p* < 0.05) Had a protective effect on the volume of the contralateral kidney (*p* < 0.01) BPF enhanced cardiac tissue strain dysfunction through elevated Pk in displacement movement (*p* < 0.01) and decreased increased time to peak (T2P) in strain movement (*p* < 0.05)	[152]
Many studies have analyzed the impact of BPF on human lipid profile	The potential synergistic effect may be due to bergamot’s ability to act on various levels, inhibiting HMG-CoA reductase, acyl-CoA cholesterol acyltransferase, and pancreatic cholesterol ester hydrolase	[153]
***Olea europaea* L. Extract**		
**Clinical Trials**	**Properties**	**Ref.**
Atherosclerotic process and plaque instability in cardiovascular disease can be improved by oleacein (10–20 μM)	Oleacein (10–20 μM) inhibited various molecular targets, including high mobility group protein 1, matrix metalloproteinase 9, matrix metalloproteinase 9–neutrophil gelatinase-associated lipocalin complex, and tissue factor	[154]
The positive impact of polyphenols on dyslipidemia in non-alcoholic fatty liver disease (NAFLD) was confirmed through a study on Olea polyphenols	Lutein may have cardioprotective properties against conditions such as coronary artery disease, atherosclerosis, and heart failure	[155]
A new therapeutic treatment approach has gained attention recently, involving capsules containing 30 percent OL to control COVID-19	This biophenol increases body temperature, respiratory rate, oxygen saturation, and blood pulse rate, resulting in shorter hospital stays by reducing erythrocyte sedimentation rate and C-reactive protein levels	[156]
The anti-inflammatory activity and potential benefits of oleic acid (OA) in reducing lipid accumulation were investigated in an in vitro model of non-alcoholic fatty liver disease (NAFLD) using McA-RH7777 cells	The OLECp has proven to be highly effective in neutralizing hydroxyl radicals and DPPH both scavenging free radicals (IC50: 2.30 ± 0.18 mg/mL) and decreasing signal area in the EPR spectra	[157]
The beneficial properties of olive oil were investigated for their effects on heart failure caused by post-myocardial infarction (PMI) induced by coronary artery constriction in rats	The myocardial redox ratio (reduced glutathione/oxidized glutathione) decreased by 44.4%, 16.4%, and 36.9% at 4 weeks postmortem interval in the LRC, LOO, and LCO groups, respectively, compared to the initial values The LRC, LOO, and LCO groups showed a significant increase in lipid hydroperoxide formation of 137.4%, 14.6%, and 97.1%, respectively, at 4 weeks PMI Rats treated with tyrosol experienced decreased infarct size and improved myocardial function compared to untreated animals	[158]
The formation of foam cells is improved by oleacein at a concentration of 50 μM	Decreased the expression of receptors (scavenger A1 receptor, CD36, lectin-like oxLDL receptor) responsible for binding and absorbing oxidized LDL into macrophages	[159]
OL at concentrations between 50 and 100 μM	Inhibited the expression of endothelial leukocytes Reduced the adhesion of monocytic cells to vascular endothelial cells	[159,160]
Comparison of health benefits of oleocanthal- and oleacein-rich extra-virgin olive oil (EVOO) with ordinary olive oil (OO) in individuals aged 40–65 with prediabetes and obesity	A significant increase in total antioxidant status and a decrease in lipid and organic peroxides compared to OO treatment (*p* < 0.05) A reduction in interferon-γ with inter-treatment differences (*p* < 0.041); weight, BMI, and blood glucose decreased significantly (*p* < 0.05) after EVOO treatment, indicating its effectiveness	[161]
Assessment of alterations in blood lipid profiles in overweight/obese subjects with slightly elevated cholesterol levels following an 8-week treatment with OLEX	Blood lipid levels did not show significant differences after 4 or 8 weeks of OLEX diet supplementation compared to placebo (*p* < 0.05) The intervention groups did not differ significantly in terms of oxLDL, glucose, blood pressure, insulin levels, or liver function parameters (*p* < 0.05). The incorporation of OLEX for 8 weeks did not result in significant modifications to blood lipid profiles	[162]

HF: heart failure, T-1095:(3-(benzo[b]furan-5-yl)-2′,6′-dihydroxy-4′-methylpropiophenone 2′-O-(6-O-methoxycarbonyl)-beta-D-glucopyranoside), SGLTs: sodium–glucose cotransporters, GLUT2: glucose transporter 2, LDL: Low density lipoprotein, MI: myocardial infarction, VAs: ventricular arrhythmias, HFpEF: heart failure with preserved ejection fraction, PCs: grape seed-derived, GSE: grape seed extract, PAECs: pulmonary artery endothelial cells, DOX: doxorubicin, RAL DOCA–salt: deoxycorticosterone acetate, BPF: bergamot polyphenol fraction, HMG-CoA reductase: 3-hydroxy-3-methylglutaryl coenzyme A, NAFLD: alcoholic fatty liver disease, COVID-19: Coronavirus Disease 19, OA: oleic acid, OLECp: extract concentrated in phenolic compounds, PMI: post-myocardial infarction, LRC: ligated regular chow, LOO: ligated olive oil, LCO: ligated corn oil, EVOO: extra-virgin olive oil, OO: ordinary olive oil, OLEX: *Olea europaea* L. leaf extract.
